# Neuroprotective effects of intranasal extracellular vesicles from human platelet concentrates supernatants in traumatic brain injury and Parkinson’s disease models

**DOI:** 10.1186/s12929-024-01072-z

**Published:** 2024-09-05

**Authors:** Liling Delila, Ouada Nebie, Nhi Thao Ngoc Le, Kelly Timmerman, Deng-Yao Lee, Yu-Wen Wu, Ming-Li Chou, Luc Buée, Szu-Yi Chou, David Blum, David Devos, Thierry Burnouf

**Affiliations:** 1 Graduate Institute of Biomedical Materials and Tissue Engineering, College of Biomedical Engineering, Taipei Medical University, 250 Wu-Xing Street, Taipei, 11031 Taiwan; 2 Univ. Lille, Inserm, CHU-Lille, U1172, Lille Neuroscience & Cognition, LiCEND COEN Center, Lille, France; 3 Alzheimer & Tauopathies, Labex DISTALZ, Lille, France; 4 International PhD Program in Biomedical Engineering, College of Biomedical Engineering, Taipei Medical University, Taipei, 11031, Taiwan; 5 NeuroTMULille, Lille Neuroscience & Cognition, Lille, France; 6 Ph.D. Program in Medical Neuroscience, College of Medical Science and Technology, Taipei Medical University and National Health Research Institute, Taipei, 11031, Taiwan; 7 NeuroTMULille, Taipei Medical University, Taipei, 11031, Taiwan; 8 Graduate Institute of Neural Regenerative Medicine, College of Medical Science and Technology, Taipei Medical University, Taipei, 11031, Taiwan; 9 Neuroscience Research Center, Taipei Medical University, Taipei, 11031 Taiwan; 10 International Master Program in Medical Neuroscience, College of Medical Science and Technology, Taipei Medical University, Taipei, 11031, Taiwan; 11 Department of Medical Pharmacology, Expert Center of Parkinson’s Disease and ALS, CHU-Lille, Lille, France; 12 International PhD Program in Cell Therapy and Regeneration Medicine, Taipei Medical University, Taipei, 11031, Taiwan; 13 PhD Program in Graduate Institute of Mind Brain and Consciousness, College of Humanities and Social Sciences, Taipei Medical University, Taipei, Taiwan; 14 Present Address: Biomedical Translation Research Center (BioTReC), Academia Sinica, Taipei, Taiwan; 15 Present Address: Institute of Clinical Medicine, National Yang-Ming Chiao Tung University, Taipei, Taiwan

**Keywords:** Exosomes, Blood, Neuroprotection, Neurological disorders, Central Nervous System

## Abstract

**Background:**

The burgeoning field of regenerative medicine has significantly advanced with recent findings on biotherapies using human platelet lysates (HPLs), derived from clinical-grade platelet concentrates (PCs), for treating brain disorders. These developments have opened new translational research avenues to explore the neuroprotective effects of platelet-extracellular vesicles (PEVs). Their potential in managing neurodegenerative conditions like traumatic brain injury (TBI) and Parkinson’s disease (PD) warrants further exploration. We aimed here to characterize the composition of a PEV preparation isolated from platelet concentrate (PC) supernatant, and determine its neuroprotective potential and neurorestorative effects in cellular and animal models of TBI and PD.

**Methods:**

We isolated PEVs from the supernatant of clinical-grade PC collected from healthy blood donors utilizing high-speed centrifugation. PEVs were characterized by biophysical, biochemical, microscopic, and LC–MS/MS proteomics methods to unveil biological functions. Their functionality was assessed in vitro using SH-SY5Y neuronal cells, LUHMES dopaminergic neurons, and BV-2 microglial cells, and in vivo by intranasal administration in a controlled cortical impact (CCI)-TBI model using 8-weeks-old male C57/BL6 mice, and in a PD model induced by MPTP in 5-month-old male C57/BL6 mice.

**Results:**

PEVs varied in size from 50 to 350 nm, predominantly around 200 nm, with concentrations ranging between 10^10^ and 10^11^/mL. They expressed specific platelet membrane markers, exhibited a lipid bilayer by cryo-electron microscopy and, importantly, showed low expression of pro-coagulant phosphatidylserine. LC–MS/MS indicated a rich composition of trophic factors, including neurotrophins, anti-inflammatory agents, neurotransmitters, and antioxidants, unveiling their multifaceted biological functions. PEVs aided in the restoration of neuronal functions in SH-SY5Y cells and demonstrated remarkable neuroprotective capabilities against erastin-induced ferroptosis in dopaminergic neurons. In microglial cells, they promoted anti-inflammatory responses, particularly under inflammatory conditions. In vivo*,* intranasally delivered PEVs showed strong anti-inflammatory effects in a TBI mouse model and conserved tyrosine hydroxylase expression of dopaminergic neurons of the substantia nigra in a PD model, leading to improved motor function.

**Conclusions:**

The potential of PEV-based therapies in neuroprotection opens new therapeutic avenues for neurodegenerative disorders. The study advocates for clinical trials to establish the efficacy of PEV-based biotherapies in neuroregenerative medicine.

**Graphical Abstract:**

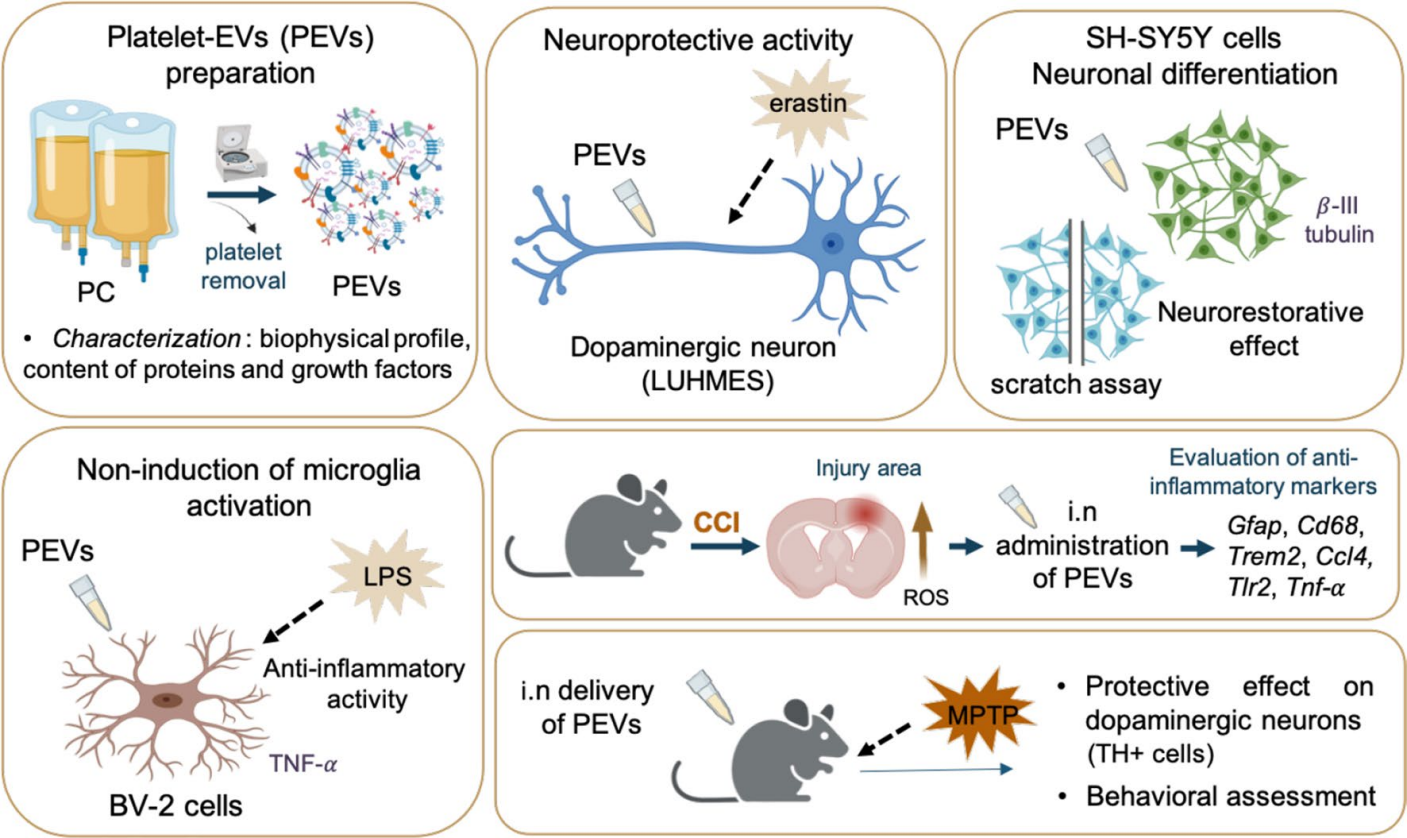

**Supplementary Information:**

The online version contains supplementary material available at 10.1186/s12929-024-01072-z.

## Background

Recent preclinical research has highlighted the therapeutic potential of human platelet lysate (HPL) in treating a spectrum of central nervous system (CNS) disorders. This notion is strongly supported by a series of preclinical studies, such as those reviewed recently [1], which highlight the protective effects of HPL in various CNS conditions. HPLs, which can be produced from human platelet concentrates (PCs) donated by healthy blood donors, have been shown to foster neuronal growth and repair in diseases, including stroke [2], Parkinson’s disease (PD) [3, 4], Alzheimer disease (AD) [5, 6], amyotrophic lateral sclerosis (ALS) [4], and traumatic brain injury (TBI) [7, 8]. These seminal works have been instrumental in establishing the groundwork for these developments. The efficacy of HPL in treating neuropathologies is thought to be routed in the unique composition of platelets, as elucidated in various publications [9–13]. Platelets harbor a complex array of growth factors, anti-inflammatory agents, cytokines, neurotransmitters, and antioxidants, primarily sourced from their granules [10, 14]. Key neurotrophic factors like brain-derived neurotrophic factor (BDNF), basic fibroblast growth factor (b-FGF), vascular endothelial growth factor (VEGF), as well as platelet factor 4 (PF4 or chemokine C-X-C motif ligand 4, CXCL4) and C–C motif ligand 5 (CCL5), also known as RANTES, (Regulated upon Activation, Normal T cell Expressed and Secreted) cytokines [15, 16], as highlighted recently [17], appear pivotal in neuroprotection, neural repair, and/or neurogenesis [1, 11–13]. Historically, the role of extracellular vesicles (EVs) in platelet function has evolved significantly since their initial identification as "platelet dust" by Peter Wolf in 1967 [18]. Today, EVs, especially  microvesicles and exosomes are recognized as key players in intercellular communication, transporting a variety of biomolecules like proteins, mRNA, and miRNA to distant cells, thus influencing a myriad of physiological processes. This has been highlighted in various studies [19–21]. Platelet-derived EVs (PEVs), in particular, which are present at high concentration in HPLs intended for neurological applications [22], carry bioactive proteins, growth factors, lipids, coagulation factors, and non-coding RNAs [23–25]. These findings raise the possibility that PEVs may themselves be a potential neuroprotective biotherapy. Thus, the therapeutic potential of HPL and PEVs in animal models presents promising implications for human CNS disorders. The observed mechanisms in these models may offer insights into similar pathologies in humans, providing a foundational basis for exploring human applications.

Our research explored here the translational potential of intranasal PEVs, especially their ability to bypass the blood–brain barrier (BBB) and exert functional impact on the brain, a crucial aspect in CNS therapy. Indeed, the pioneering studies of Kodali et al. [26] on mesenchymal stromal cell-derived EVs in TBI models, and the internalization of PEVs by human brain endothelial cells [27], underscore the possible significance of this approach. The primary objective of our study was thus to isolate and analyze a specific type of PEVs that can be readily isolated from clinical-grade human PC donated by healthy donors, and to unveil their potential roles in neuroprotection and neuroregeneration in TBI and PD models. The choice of animal models for our study was made with careful consideration of their established relevance and validity in the field. For investigating TBI, we employed the controlled cortical impact (CCI) model. CCI is widely recognized as a well-established pre-clinical method for mimicking TBI in humans [28] due to its ability to replicate the mechanical aspects of brain injury and the subsequent pathophysiological processes, including inflammation. Our choice of the CCI model is reinforced by our prior studies, wherein we successfully demonstrated the efficacy of intranasally administered platelet lysate, referred to as HPPL, in modulating the effects of TBI [7, 8]. Similarly, for PD research, we utilized the MPTP-induced model [29], which reliably simulates PD-like degeneration of dopaminergic neurons and resembles the pathological progression seen in humans. We have shown the suitability of this model for exploring the neuroprotective effects of the intranasal HPPL [9]. The extensive use and validation of both the CCI and MPTP models in our previous research provides a strong foundation for our studies, which opens new avenues for non-invasive translatable biotherapeutic strategies in brain health and disease management.

## Materials and methods

### Platelet concentrates (PCs) collection

Allogeneic PCs were collected using apheresis with a MCS + platelet collection system (Haemonetics, Braintree, MA, USA) from volunteer regular healthy donors at the Taipei Blood Center (Guandu, Taiwan), with approval from the Institutional Review Board of Taipei Medical University (TMU-JIRB N201802052). These clinical-grade PCs, initially intended for transfusion, were suspended in 100% plasma, were not leucoreduced, and were anticoagulated with a citrate–phosphate-dextrose solution. They were stored at 22 ± 2 °C under mild agitation, following standard licensed procedures. Upon reaching their expiry date, five days after collection, the PCs were transported to the Taipei Medical University laboratory within 90 min under controlled ambient conditions. Upon arrival, the PCs were placed on a slow-speed platelet agitator at 22 ± 2 °C and processed either the same day or the next. Before processing, the sample was collected aseptically to determine the blood cell count using a ABC Vet blood cell counter from ABC Diagnostics (Montpellier, France).

### Preparation of PEVs

**Fig. 1:**
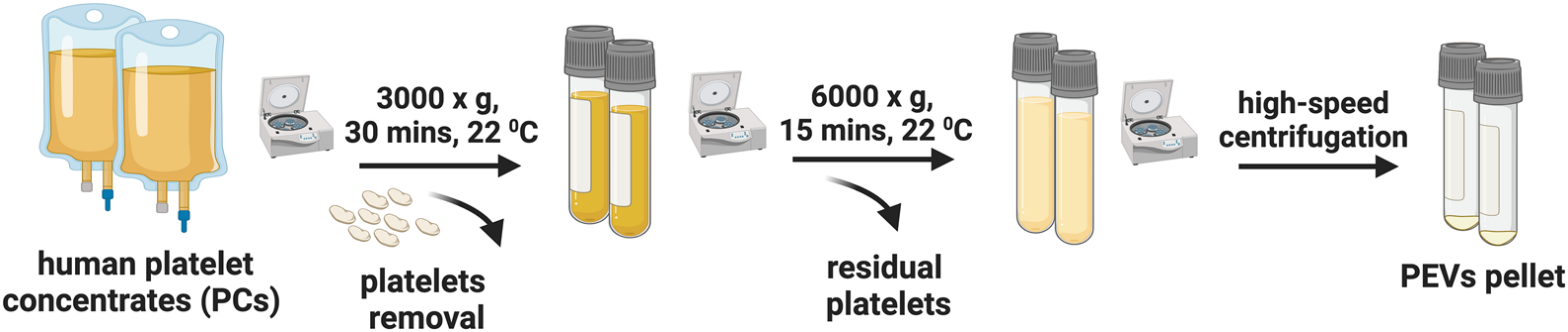
Preparation of PEVs. Diagram illustrating the process of preparing PEVs. PC was centrifuged at 3000×*g* for 30 min to pelletize the platelets and obtain PC supernatant followed by 6000×*g* for 30 min to remove the residual platelets. PEVs were isolated by high-speed centrifugation at 25,000×*g* for 90 min and resuspended in PBS. PCs: Platelet concentrates; PEVs: Platelet-extracellular vesicles

PEVs were prepared from at least three PC donations (n = 3) and were later pooled for analysis. PEVs were obtained from the plasma supernatant of PC, as previously described [30] (Fig. 1). Firstly, PC was centrifuged at 3000×*g* for 30 min to pelletize the platelets. The supernatant underwent a second centrifugation at 6000×*g* for 10 min at 25 ± 2 °C to eliminate any residual cell debris. PEVs were isolated through high-speed centrifugation at 25,000×*g* for 90 min at 18 ± 2 °C. The recovered PEV pellet was washed, re-suspended in phosphate buffer saline (PBS) at a ratio of 0.01 mL per mL of the original PC volume, aliquoted, and stored at − 80 °C until further use.

### Biophysical and membrane markers characterization of PEVs

Dynamic Light Scattering (DLS): We used 100 µL samples for DLS size distribution measurements in a disposable low-volume cuvette, as described previously [22, 30]. Measurements were made using a Zetasizer Nano ZS from Malvern Instruments, UK.

Nanoparticle Tracking Analysis (NTA): NTA (NanoSight NS300, Malvern Instruments, UK) was used. PEVs were diluted 10,000-fold in 0.1 µm-filtered PBS. An 800 µL sample was injected via a syringe pump and data was collected over 60 s.

Tunable resistive pulse sensing (TRPS): TRPS analysis was done by qNano (Izon, New Zealand) with 400 nm pore size of nanopore membranes and 210 nm (CPC200) particle size calibrating beads. 40 µL of pre-diluted PEVs (10-fold) were introduced to the upper fluid cells. The particles rate was recorded for 10 min. The data were processed by qNano software.

PEV surface markers: The EV markers were detected using a Exo-Check Exosome antibody membrane array (EXORAY210B-8) from System Biosciences, following the manufacturer's instructions. 50 µg of PEVs sample was treated with lysis buffer, followed by the addition of 1 µL of labeling reagent, and a 30-min incubation with continuous mixing. Any excess labeling reagent was removed by using a provided separation column and several washing steps. After, 5 mL blocking buffer was added. The labelled PEVs lysate/blocking buffer mixture then was introduced to an antibody array membrane and incubated overnight at 4 °C on a shaker. Subsequently, the membrane was washed to eliminate free molecules and followed by the incubation of the detection reagent mixture for 30 min at room temperature. Following this step, streptavidin-HRP and chemiluminescent detecting solutions were added. Each location emitted light corresponding to the presence of antibodies bound to their respective immobilized capture sites on the membrane, which then was imaged using an Invitrogen iBright CL750 system. The blank served as an internal control. To quantify the intensity of the antibody-bound bands, the background intensity of the blank was subtracted from each band’s intensity.

Cryo-electron microscopy (cryo-EM): Samples were prepared for cryo-EM on a FEI Vitrobot by pipetting 4 μL onto 200 mesh holey carbon film with glow-discharged 20 s (Electron Microscopy Sciences), blotted for 3 s in 100% humidity at 4 °C, and stored in liquid nitrogen until imaging. Images were taken at 50,000-fold magnification a dose of 2417 e/nm^2^s operating at 200 kV on a FEI Tecnai F20 at Cryo-EM facility, Academia Sinica (Nangang, Taiwan).

### Proteins, growth factors quantification, proteomics, and bioinformatic analysis

The total protein content was measured using a bicinchoninic acid (BCA) assay. PEVs was initially diluted 10-fold. A 25-µL sample then was mixed with 200 µL of BCA working reagent (1:50 ratio) in a 96-well plate and shaken for 30 s. After incubating for 30 min at 37 °C, the purple color that developed was measured at 562 nm. Bovine serum albumin (BSA) was used as a standard, with concentrations ranging from 25 to 2000 µg/mL.

The levels of growth factors present in PEVs were quantified in triplicate using a DuoSet ELISA kit, following the manufacturer's instructions (R&D Systems, DY248, DY222, DY795, DY236, DY293, Minneapolis, MN, USA), as done previously [31]. PEVs were diluted 1000-, 10-, and 5-fold to determine the concentrations of PF4, PDGF-AB, and BDNF, respectively. EGF and VEGF were measured without dilution. Absorbance was measured and analyzed with a BioTek EPOCH 2 microplate reader (Santa Clara, CA, USA).

The Liquid Chromatography-Tandem Mass Spectrometry (LC–MS/MS) procedure for proteomic analysis was performed as previously described [7]. PEVs samples were treated overnight with acetone pre-cooled to − 20 °C at a sample-to-acetone volume ratio of 1:4. The mixture was then centrifuged at 15,000×*g* for 10 min at 4 °C. The resulting pellet was washed twice using a 1:4 ratio of cold acetone to water, followed by centrifugation at 13,000×*g* for 10 min at 4 °C. The supernatant was discarded, and the pellet was air-dried and re-suspended in 6 M urea. Protein content was quantified using the BCA protein assay, and 20 μg of protein was used. Data Analysis: For each raw data file generated by MS, peak lists were prepared using Data Analysis version 4.3 (LC-QTOF; Bruker Daltonics, Billerica, MA, USA) and Proteome Discoverer versions 2.2 or 2.4 (LTQ Orbitrap; ThermoFisher Scientific). An human UniProt Swiss-Prot database (comprising 20,431 annotated proteins, release 2019.07) was used for analysis. The false discovery rate (FDR) for spectrum and protein matching was set at 1%. We have included HPPL sample in this proteomic analysis simultaneously with PEVs to assess the similarity of their protein lists. Furthermore, the PEVs proteins were identified using Vesiclepedia and ExoCarta, the two main manually curated databases that compile identified EV cargo. The functional enrichment analyses were performed using an open-access software, Functional Enrichment analysis tool (FunRich, FunRich version 3.1.4).

For western blot (WB) analysis, 30 mg of PEVs’ protein were suspended in Bio-rad LDS samples buffer 4X and heated for 5 min at 100 °C. Samples were loaded onto GenScript 12% SDS-PAGE gels and separated for 30 min at 200 V. The proteins were then electrotransferred for 50 min at 100 V onto a hydrophobic polyvinylidene difluoride membrane (PVDF) (Pall, USA). After 45 min of blocking in 5% BSA, the membranes were incubated with the primary antibody for an additional night at 4 ± 2 °C. The monoclonal antibodies were used as follow: anti-rabbit CD41 (1/1000, ab134131, Abcam), anti-rabbit CD61 (1/1000, ab7166, Abcam), anti-rabbit CD42a (1/1000, ab133573, Abcam), CD62P (1/1000, ab255822, Abcam), anti-mouse CD9 (1/500, sc-13118, Santa cruz), and anti-mouse CD63 (1/500, sc-5275, Santa cruz). The membranes were then washed three times with 1 × Tris-NaCl-Tween-20 (TNT) for 15 min before the incubation with horseradish peroxidase (HRP)-labeled secondary antibodies for 45 min at room temperature. Immuno-reactivity was detected using the ECL kit (RPN2106, GE Healthcare), and visualized with a UVP system (Level, 115 V ~ 60 Hz, Upland CA, USA).

### Procoagulant assays

Microparticle (MP)-activity assay: The assessment of pro-thrombogenic activity associated with the presence of PEVs expressing functional PS was conducted using an Zymuphen MP-activity assay (Hyphen BioMed, Paris, France), as previously described [31]. 100 µL of PEVs were pre-diluted at 2 × 10^3^ and 3 × 10^4^-fold respectively, in the sample diluent. These were then added to an Annexin-precoated microplate and incubated for 60 min at 37 °C. Activation of prothrombin to thrombin was initiated by adding 100 µL of factor Xa-Va in combination with calcium, and 50 µL of prothrombin, followed by a 10-min incubation at 37 °C. After five washing steps with 300 µL of a washing solution, 50 µL of a chromogenic substrate was introduced. The subsequent formation of a chromogenic substance following a 3-min incubation at 37 °C was halted by addition of 50 µL of 2% citric acid. Absorbance was then measured at 405 nm. Platelet pellet lysate (PPL) and its heat treated fraction (heated-PPL or HPPL), which are prepared from isolated platelets and are enriched in platelet factors and contain PEVs [9, 31], were included as controls using the same volume as the PEVs.

STA-procoagulant-phospholipid assay-(PPL): The STA-PPL assay (Diagnostica, Stago, Asnières, France) was used to evaluate the procoagulant activity associated with PEVs, as described [32]. Undiluted PEVs, PPL and HPPL (25 µL) were combined with 25 µL of citrated human plasma depleted of phospholipids. This mixture was incubated at 22 ± 2 °C for 120 s. The coagulation process was triggered upon adding 100 µL of activated factor X (FXa), and the clotting time was then recorded using an STA compact automatic coagulometer. Both positive and negative controls, as provided by the kit, were tested following the manufacturer's guidelines.

### Assessment of neuroprotective activity of PEVs in vitro in LUHMES cells

LUHMES cell culture and treatment: LUHMES cells were seeded at a density of 4 × 10^6^ in a flask pre-coated with 50 μg/mL poly-l-ornithine (P3655, Sigma-Aldrich) and 1 μg/mL fibronectin (F1141, Sigma-Aldrich). They were cultured in Advanced DMEM/F12 medium (12634010, Thermo Fisher), supplemented with N2 (17502-048, Thermo Fisher), 2 mM l-glutamine (25030-124, Thermo Fisher), and 40 ng/mL recombinant bFGF (4114-TC, R&D Systems). The cells were incubated at 37 °C in a humidified atmosphere with 5% CO_2_ to facilitate proliferation. For differentiation, 2.5 × 10^6^ cells were seeded in a T75 flask containing the proliferation medium and incubated for 24 h. The medium was then switched to a differentiation medium, composed of Advanced DMEM/F12, N2 supplement, 2 mM l-glutamine, 1 mM dibutyryl-cAMP (D0627, Sigma-Aldrich), 1 μg/mL tetracycline (T-7660, Sigma-Aldrich), and 2 ng/mL recombinant human BDNF (DY248, R&D Systems). After two days, the cells were transferred to 24-well plates to complete the differentiation process over an additional three days. On the fifth day of differentiation, LUHMES were treated with 5% (v/v) PEVs and, subsequently, a dose of 1 µM erastin (E7781, Sigma-Aldrich) was added for neurotoxic stimulation through induction of ferroptosis. Cell viability was evaluated 24 h later.

Cell viability assay: 50 μL of CCK-8 solution (96992, Sigma-Aldrich) containing WST-8 was added to each well containing 500 μL of cell medium. The amount of formazan produced is proportional to the number of viable cells. The plate was incubated for 4 h at 37 ± 1 °C in a 5% CO_2_ humidified incubator. After incubation, absorbance at 450 nm was measured using a Tecan Infinity M200 microplate reader. Viability was calculated as a percentage of untreated control cells, using the formula: [(O.D. of sample − O.D. of blank)/(O.D. of control − O.D. of blank)] × 100.

### PEVs impact on differentiation and restoration of human SH-SY5Y neuroblastoma cells

SH-SY5Y cells were grown in DMEM (11965092, Thermo Fisher) supplemented with 10% fetal bovine serum (FBS), and 100 U/mL penicillin. The cells were incubated at 37 ± 1 °C in a humidified 5% CO_2_ atmosphere. They were kept in T75 flasks until reaching 80–90% confluence. For sub-culturing, the cells were washed with PBS, treated with trypsin–EDTA for 2–3 min until detached, and then re-suspended in fresh medium. Depending on the experiment, they were either re-seeded in T75 flasks or plated at a specific density. To determine the ability of PEVs to stimulate cell maturation, undifferentiated SH-SY5Y cells were seeded at 24 well-plate and after one day of incubation, they were treated using 10 μM retinoic acid (RA, RM2625, Sigma-Aldrich) as a differentiation agent, 2% (v/v) PEVs, or 2% (v/v) HPPL. RA and HPPL served as a positive control and some cells were maintained in a medium containing 0.5% FBS without any treatment, used as a negative control group. The culture medium was refreshed every three days, and the cells were examined on the seventh day of the experiment. To assess the promotion of SH-SY5Y cell differentiation, fluorescence labelling with β-III tubulin (Ab18207, Abcam, 54 kDa, 1:500 dilution) was used. The culture medium was removed, and the cells were rinsed with PBS before fixation in 2% paraformaldehyde (PFA), at RT for 30 min. Following this, the cells were permeabilized for 20 min at RT using 0.2% PBS-Triton X-100, and non-specific binding was blocked for 1 h with 1% BSA in PBS. The primary anti-β-III tubulin (Ab18207, Abcam) was added and incubated overnight at 4 °C. After, the wells were washed with PBS, and the cells were incubated for 1 h with Alexa Fluor-488-conjugated goat anti-rabbit IgG antibody (A32723, Invitrogen), the nuclei were stained using DAPI. Fluorescence images were captured using a Leica DMi8 fluorescence microscope (Sage Vision, West Chester, PA, USA). The fluorescence intensity of β-III tubulin was quantified using Image J software (1.6, NIH, Bethesda, MA, USA) by measuring the integrated fluorescence density for each treatment in three separate trials. Furthermore, the quantification of neurite extensions was done by FIJI plugin Simple Neurite Tracer (SNT. V4.2.1). From the captured images of B-III tubulin fluorescence, the length of each neurite extension was measured individually (Figure S1). The ratio of neurite length in treated cells to that in untreated cells was then calculated. Moreover, to investigate the neurorestorative properties of PEVs, a scratch assay was conducted on SH-SY5Y cells that were differentiated by RA treatment. Following this, a 100 µL tip was used to create a wound in the cell monolayer. Subsequently, we added 5% (v/v) of PEVs and HPPL at the same dose as a positive control. The wounded zone free of cells was examined under microscopy (Leica DMi8 microscope, Wetzlar, Germany) over two days. The results are expressed as a wound-healing index, determined by the formula: (initial wound area − final wound area)/initial wound area.

### Assessment of anti-inflammatory activity of PEVs in BV-2 microglia cells

BV-2 microglia cell culture: We used immortalized mouse microglial BV-2 cells to assess the ability of PEVs to modulate microglial activation. BV-2 cells were cultured in T75 flasks using DMEM medium (SH30243, Thermo Fisher) supplemented with 10% FBS and 100 U/mL of penicillin. The cells were incubated at a controlled temperature of 37 ± 1 °C in a 5% CO_2_ humidified incubator. When the cell confluence reached 80–90%, the cells were passaged. A total of 2 × 10^4^ cells were grown per well in 24-well plates to be used for our experiments. The cells were supplemented with 10% FBS until confluent. Then, they were pre-treated with PEVs at a concentration of 5% (v/v) medium and 5% (v/v) HPPL, which was used as a non-inflammatory control. After 1 h, 100 ng/mL of lipopolysaccharide (LPS, L4130, Sigma-Aldrich) was added to trigger inflammation. The cells lysate and supernatant were collected after a 24-h incubation period. The upregulation of *Tnf*-$\alpha$ gene expression was assessed in the cell lysate by using quantitative reverse transcription PCR (RT- qPCR). Mouse TNF-α (DY410, R&D System) IL-6, and IL-1β ELISA kits (431304, 432604, BioLegend) were used following the manufacturer's instructions to determine the levels of inflammatory markers in the cell supernatant.

### Assessment of neuroprotective activity of PEVs in TBI and PD animal models

Study of PEVs diffusion in the mice brain: PEVs, as well as HPPL used as a control, were labelled with Alexa Fluor 568 dye (Thermo Fisher Scientific, San Jose, CA, USA) following the supplier's instructions and as described in our recent study [33]. A total volume of 60 μL of fluorescently-labelled PEVs or HPPL were administered intranasally, 2–3 μL at a time, alternating the nostrils. The mice were anaesthetized 7 h after the final administration using Zoletil-50 (66F4, Virbac, France) and Rompun (PP1523, Bayer, Switzerland) and perfused with 0.9% cold NaCl. The brains were subsequently fixed in 4% PFA and transferred to a cryoprotective solution. Sagittal cryosections with 30 μm thickness were prepared and imaged using a fluorescent slide scanner (ImageXpress® Pico) for observation.

Ethical approval for animal study of CCI mouse model of TBI: The study adhered to ethical guidelines for the welfare of animals and were conducted following the animal use protocol from Taipei Medical University (TMU, Taipei, Taiwan; application no. LAC 2020-0042). Male C57/BL6 mice (8-week-old, 20–30 g weight) were obtained from the Taiwan National Laboratory Animal Center (Nangang, Taipei, Taiwan). The mice were kept in groups of 4–5 per cage (cage size: L29.3 × W18.9 × H12.9 cm ± 3%) in a controlled environment with constant temperature (19–24 °C) and humidity (60–70%) on a 12-h light/dark cycle at TMU Laboratory Animal Center.

CCI method: The mice were randomly allocated into three groups: a sham group, and two treatment groups receiving PEVs and PBS (as a control, n = 7–10 mice/group). *Anesthesia and Surgery:* Mice were anesthetized via intraperitoneal injection with a mixture of Zoletil (10 mg/kg) and xylazine (10 mg/kg) given at a dosage of 10 μL/g body weight. Following anesthesia, the surgical area was shaved, and the mouse was placed in a stereotaxic device with its head immobilized by ear bars. Surgical sites were cleansed by using cotton tips soaked in iodine and ethanol. A midline incision exposed the skull, where a 4-mm diameter hole was made between the bregma and lambda on the right hemisphere. Mild injury was inflicted using an impactor (eCCI-6.3, Custom Design & Fabrication, Sandston, VA, USA) with specific parameters (3-mm tip at a velocity of the actuator of 3 m/s, a deformation depth of 0.2 mm, and a dwell time of 250 ms) as before [7]. The injury was initiated by hitting the surface of the cortex perpendicularly. Sham-operated (Sham) mice underwent a hole-drilling procedure without any brain impaction. The wound was then closed, and antibiotic ointment was applied. Mice were placed in a heated cage for recovery. *Treatment Protocol:* Approximately 2 h post-injury, samples of 60 μL or 1.2 × 10^8^ number of PEVs and PBS (as a control) were administered intranasally using a pipette by alternating the nostrils (2-3-μl at a time per nostril)﻿ and maintaining 5-min intervals between each 20-μL administration. This treatment was repeated on 3 consecutive days, with each mouse receiving 180 μL in total. On day 7, mice were sacrificed by cervical dislocation, the brains were quickly collected and rinsed in cold PBS, and the injured area of the ipsilateral cortex was collected using a 4.0-mm biopsy punch. Samples were then frozen in liquid nitrogen until further gene expression analysis by RT-qPCR.

Genes expressions analysis: RNA extraction from the collected tissues was performed using a RNeasy Lipid Tissue Mini Kit (cat. no. 74804, Qiagen) following the manufacturer's protocol. 1000 μL of Qiazol was added to each frozen sample and homogenized using a tissue ruptor. After 5 min, 200 μL of chloroform was added and shaken vigorously. The mixture was centrifuged at 12,000×*g*, for 15 min at 4 °C, then, the upper aqueous phase containing the RNA was transferred to a new tube, and an equal volume of 70% ethanol was added and vortexed. 700 μL of each sample was next assigned to a RNeasy Mini Spin column sitting on a 2-mL collection tube and centrifuged at 8000×*g* for 15 s. The flow-through was discarded, and the spin column was put back on the tube. Then 700 μL of RW1 was added to the spin column and centrifuged at 8000×*g* for 15 s. Again, the flow-through was discarded, and 500 μL of RPE was added to the spin column. The column was centrifuged at 8000×*g* for 15 s. The flow-through was then discarded, and this step was repeated. Finally, the spin column was transferred to a new 1.5 mL Eppendorf tube, and the total RNA was eluted by adding 50 μL of RNase-free water and centrifugation at 8000×*g* for 1 min. NanoDrop2000 (Thermo Fisher Scientific, Waltham, MA, USA) was next used to quantify the total RNA concentration. *RT-qPCR for inflammatory markers and oxidative stress.* We used 1µg of total RNA to synthesize a complementary DNA (cDNA) using an Applied Biosystems High-Capacity cDNA reverse transcription kit (ref 4368814). Afterward, the RT was run with the following program at a thermal cycler (StepOneTM Real-Time PCR System): 10 min at 25 °C, 120 min at 37 °C, and 85 °C, 5 min. The obtained cDNA was stored at − 20 °C before using in qPCR. Validated primers (supplementary Table 1) were used to perform qPCR. Reactions were prepared using 5 μL of Power SYBR Green PCR Master Mix (cat.4367659, ThermoFisher Scientific, Watham, MA, USA), 0.1 μL of forwarding primer, 0.1 μL of reverse primer, 2 μL of cDNA pre-diluted 20 times, and 2.8 μL of RNase-free water for each sample. StepOneTM Real-Time PCR System (ThermoFisher Scientific) was used with an amplification profile of 50 °C, 2 min, 95 °C for 10 min, followed by 40 cycles of (95 °C for 15 s, 60 °C, 25 s, 95 °C for 15 s), melting curve at 60 °C for 1 min. Inflammatory markers targeting astrocytic markers, microglial markers, cytokines and chemokines, and chemokines receptors, including *Gfap*, *Cd68*, *Trem2*, *Ccl4, Tlr2*, and *Tnf-*$\alpha$, were screened.

MPTP mice model of PD: All experiments were authorized by the National Ethical Committee in Animal Experimentation (Comité d'éthique en Expérimentation Animale Nord-Pas de Calais CEEA no.75) as well as the French Ministry of Education and Research (agreement number: 2018060818218219 v4) and were carried out in strict accordance with European Union Directive 2010/63/EU. The research was reported in compliance with the ARRIVE criteria for reporting animal experiments. 5-month-old male C57BL/6 mice (Janvier Le Genest St Isle, France) with a weight between 26 and 30 g from were used. The mice were housed in the animal laboratory of Département de Pharmacologie Médicale, University of Lille, France, with 9–10 mice of each group in large cages, at a temperature of 19–24 °C, 60–70% humidity, on a 12-h light/dark cycle. The animals were given a 7-day period to acclimate to the laboratory environment prior to conducting any experiments. They were randomly divided into 4 groups: sham group (n = 7) and MPTP groups with at least 17 mice in each. MPTP/PBS group was used as control. PEVs at the number 4 × 10^10^ was delivered intranasally approximately 15 h before MPTP intoxication. Mice were injected with 20 mg/kg (MPTP) (reference 199915, Sigma-Aldrich) or received saline by intraperitoneal injection at the day 0 (sham). The open field behavior test was performed at day 7 and after the mice were sacrified, and the brain tissues were collected for further analysis.

Open field test: The open field test was utilized in conjunction with an open-field infrared actimeter to evaluate spontaneous locomotor activity. Actimetry device (Panlab, Barcelona, Spain), an open field apparatus made from transparent Plexiglas a with a size (45 cm × 45 cm × 35 cm) integrated with two frames of infrared beams, was used. This test is based on mice natural tendency to explore a new environment. The activity was recorded over a 10-min. The major result was the total distance walked (in cm), mean velocity and rearing numbers, which were captured by two rows of infrared photocells and analyzed using the Actitrack software.

Mice brain tissue preparation for immunohistochemistry (IHC): Mice were anesthetized by dolethal via intraperitoneal injection (200 mg/kg), and perfused intracardially with cold saline containing heparin (5 mg/mL), followed by 4% PFA. Whole mice brain was collected and immediately stored in PFA overnight at 4 °C. After, they were transferred into 30% sucrose overnight before freezing at − 80 °C for cryo-sectioning. Frozen brains were cut in coronal sections with 20 μm of thickness and collected in coated glass slides.

Tyrosine hydroxylase (TH) staining: IHC to stain the TH marker of dopaminergic neurons was performed as described below. Substantia nigra (SN) were incubated with rabbit polyclonal anti-TH antibody (1/1000, AB152, Merck Millipore) overnight, followed by anti-rabbit goat secondary antibody (1/500, BA-1000, Vector), and avidin-biotinylated horseradish peroxidase HRP complex (PK*-*6100, Vectastain, Elite), and 3,3’Kdiaminobenzidine (DAB). Slices were imaged on a multi slide scanning microscope (ZEISS Axio Scan Z.I slide scanner (20× objective) and Zen software Blue edition (Zeiss, Oberkochen, Germany). The total numbers of TH-stained neurons in SN were bilaterally counted in seven sections between bregma – 2.92 mm and – 3.88 mm. For each animal, the neuron counts in each section were summed.

### Statistical analysis

Statistical analyses were performed using GraphPad Prism software version 10.2.3 (La Jolla, CA, USA), and data are expressed as a mean ± standard deviation (SD) or standard error of the mean (SEM). Following confirmation of normal distribution, a one-way analysis of variance (ANOVA) followed by Fisher’s least significant difference (LSD) test was performed for comparison, and differences were considered significant at *p* < 0.05. Number of independent experiments is described in the figure legends.

## Results

### Bio-physical, biochemical, and functional characterization of PEVs

**Fig. 2:**
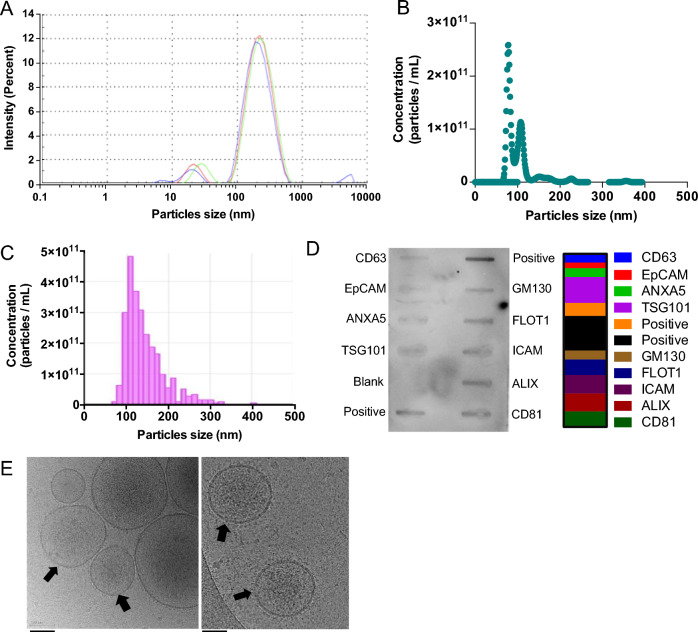
The bio-physical characterization of PEVs. **A** The size population profile of PEVs is determined by DLS. **B** PEVs number and size distribution determined by TRPS. The sample was diluted 10-fold in 0.1 μm-filtered PBS prior to analysis. **C** PEVs number and size distribution determined by NTA. The sample was diluted 100-fold in 0.1 μm-filtered PBS prior to the analysis. **D** Images displaying human-specific antibody arrays after incubation with PEVs. 50 μg proteins were loaded into an array. The quantification of the intensity of emitted light corresponding to the presence of bound antibodies to the membrane was displayed (right panel) assessing EVs markers TSG101, ALIX, CD81, FLOT1, CD63, and ANXA5. Cell adhesion protein markers ICAM, EpCAM, and Golgi marker GM130. **E** Representative CryoEM image displaying a lipid bilayer membrane in PEVs, indicated by a black arrow. Scale bar = 100 nm. PC: Platelet concentrate; PEVs: Platelet-extracellular vesicles

We evaluated PEVs product obtained from 25,000×*g* ultracentrifugation of platelet-concentrate supernatants using a variety of techniques, including DLS, NTA, and TRPS, to determine their size and content. Furthermore, we performed Exo-Check Exosome Antibody testing to assess the presence of common EV markers and cryo-EM to visualize the structural features of PEVs. The isolated PEVs comprised a main population ranging from 70 to 350 nm, with the predominant size, as determined by DLS, of approximately 200 nm. A minor population of events with a mean size of 20 nm was also detected. The concentrations of the PEVs ranged between 10^10^ and 10^11^ particles/mL, with the main size distribution spanning from 100 to 275 nm, as revealed by NTA. These findings were further supported by TRPS analysis using a 400 nm nanopore membrane, which indicated that the main size of the PEVs ranged from 150 to 300 nm (Fig. 2A–C). PEVs expressed the expected EV markers with the highest intensity detected on the membrane array as follows: TSG101, ALIX, CD81, FLOT1, CD63, and ANXA5. Expression of proteins related to cell adhesion, including ICAM, EpCAM, and the Golgi marker GM130 were also detected (Fig. 2D). The WB analysis demonstrated that PEVs carry platelet-associated glycoproteins, including P-selectin (CD62p), GPIIb/IIIa (CD41/CD61), and GPIX (CD42a), as well as confirming the presence of CD63 and the additional detection of CD9 (Figure S2). Cryo-EM analysis revealed particles enveloped by a lipid bilayer, with particles diameters in the range of 50 to 300 nm (Fig. 2E).

### Proteomic analysis of PEVs

**Fig. 3:**
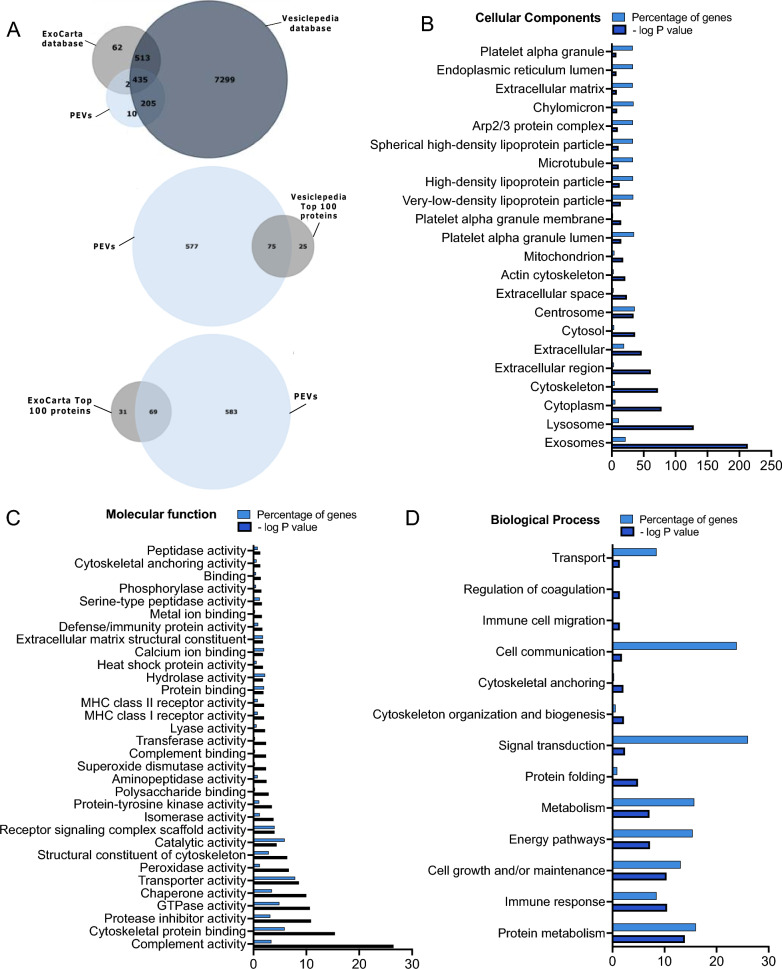
Proteomic analysis of PEVs. **A** Venn diagram showing the number of PEVs proteins that are found in ExoCarta and Vesiclepedia databases and the presence of 72 and 69 PEVs proteins among the top 100 detected in Vesiclepedia and Exo-carta, respectively. **B** The visualized results of representative functional enrichment used FunRich analytical tool to of the 652 PEVs proteins on the cellular component **C** Molecular function and **D** Biological processes. Each bar is proportional to the percentage of genes. The significance cutoff was p < 0.5. PEVs: Platelet-extracellular vesicles

A comprehensive proteomic analysis was performed using LC–MS/MS to profile the protein content in PEVs. A total of 652 proteins (supplementary Table 2) with a protein level FDR of less than 1%, were detected in PEVs. Among the 652 proteins, 640 (98.1%) and 437 (67%) are listed in the Vesiclepedia and ExoCarta databases, respectively (Fig. 3A). Furthermore, PEVs contained a considerable number (69 and 72, respectively) of the top 100 most reported proteins in exosomes and EVs, according to ExoCarta and Vesiclepedia. Our analysis indicated a predominance of proteins in PEVs, aligning with several categories stated by the Minimal Information for Studies of Extracellular Vesicles (MISEV) 2018 and 2023, as shown in Supplementary Table 3. Notably, we found non-tissue-specific transmembrane proteins, such as CD63, CD81, and CD9 (category 1a), and platelet proteins like CD41 and CD42a, as detected by WB (category 1b). In category 2a, we observed cytosolic proteins with lipid or membrane protein-binding capabilities, including ALIX, Annexins, and EDH proteins. Category 2b included proteins associated with heat shock protein HSP70, cytoskeleton, actin, and tubulin. Additionally, mitochondrial enzymes SOD1 and SOD2, endoplasmic reticulum-associated proteins, and the actin-binding protein ACTN1/4 (category 4a) were present in PEVs. Functional components, such as cytokines, growth factors, antioxidants, adhesion proteins, and extracellular matrix proteins (categories 5a-b) were also identified. FunRich analytic tool further elucidated the cellular component, molecular function and biological processes of the PEVs proteins. Exosomes, lysosome, cytoplasm, cytoskeleton, and platelet alpha granules were among the most abundant components, as shown in Fig. 3B. The functional annotation analysis identified, as highly enriched cluster related to GTPase activity, cytoskeletal binding, protease inhibitor, catalytic activity, transporter activity, protein-tyrosine kinase activity, and SOD (Fig. 3C). The examination of biological processes associated with PEVs encompassed cell communication, signal transduction, cell growth, and maintenance (Fig. 3D).

### Total protein, pro-coagulant assays, and growth factors in PEVs

The protein concentration of the PEVs fraction was determined using the BCA assay, with the average total protein concentration ranging from 3 to 4 mg/mL. To further characterize the PEVs, we performed assays to confirm their low procoagulant activities, given the known role of platelets in hemostasis. PS, a pro-coagulant phospholipid expressed by activated platelets and potentially by PEVs, was less prevalent in PEVs compared to the PPL (p < 0.001) and HPPL (p < 0.0001) fractions, as shown in Figure S3.A. The STA-procoagulant-phospholipid assay (Figure S3.B), which specifically measures the impact of PEVs in promoting blood coagulation revealed that the coagulation time induced by PEVs (approximately 14 s) was shorter than that induced by HPPL (ca. 24 s; p < 0.001), but not significantly different from that by PPL (ca. 13 s). We also quantified selected platelet growth factors in PEVs using ELISA assays. The results (N = 3) indicated measurable amounts of PF4, BDNF, PDGF, EGF, and VEGF (expressed in ng/mL) of approximately 311 ± 40; 8.2 ± 0.9; 11.9 ± 1.6; 0.51 ± 0.08, and 0.025 ± 0.021, respectively.

### In vitro neuroprotective activity of PEVs on LUHMES cells exposed to the erastin neurotoxin

**Fig. 4:**
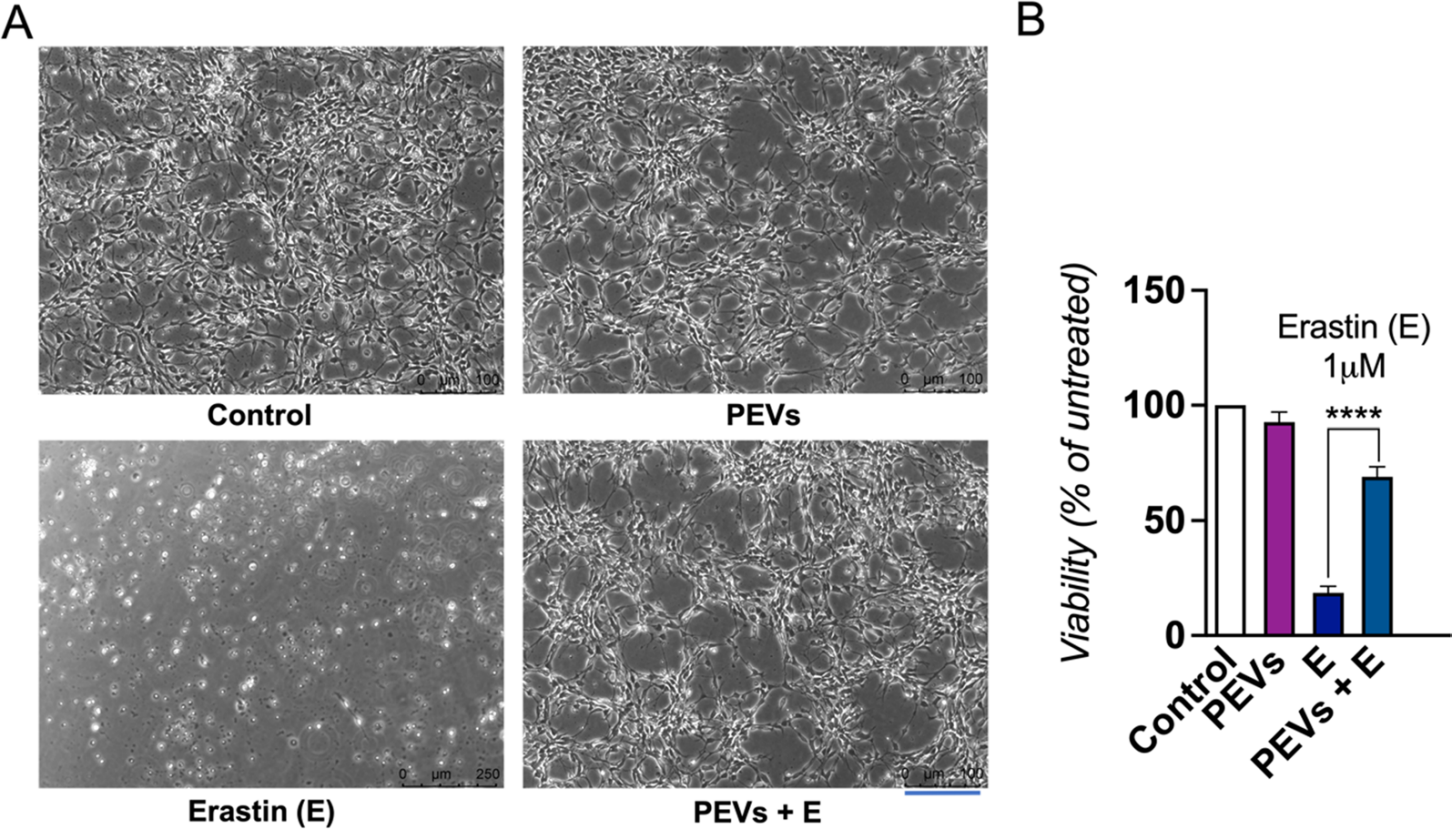
The effect of PEVs treatment on the viability of LUHMES cells exposed to erastin. **A** LUHMES were treated with PEVs and subsequently, a dose of 1 µM neurotoxin erastin was added. Images showing the cell morphology after 24 h. The scale bar is 250 μm. **B** LUHMES cell viability after 24 h was quantified by a CCK-8 assay. LUHMES were exposed or not to erastin. N = 3, ****p < 0.0001 as compared to the control with erastin, a ferroptosis promoter. PEVs: Platelet-extracellular vesicles; E: Erastin

LUHMES cells were pre-treated with PEVs for 1 h prior to exposure to 1 μM erastin. Our observations indicated that the LUHMES cells treated with PEVs maintained normal cellular morphology. This finding contrasted with the altered morphology observed by cells exposed to the pro-ferroptosis compound erastin alone, as depicted in Fig. 4A. To assess cell viability post-erastin exposure, we conducted a CCK-8 assay 24 h later, as presented in Fig. 4B. The treatment with PEVs resulted in substantial neuroprotection against erastin-induced toxicity. Specifically, while the cell viability was reduced to 17% by erastin alone, it was significantly higher (p < 0.0001) in the presence of PEVs, reaching 71% of the control.

### In vitro neuromaturation and neurorestorative effect on PEVs on SH-SY5Y neuroblastoma cell line

**Fig. 5:**
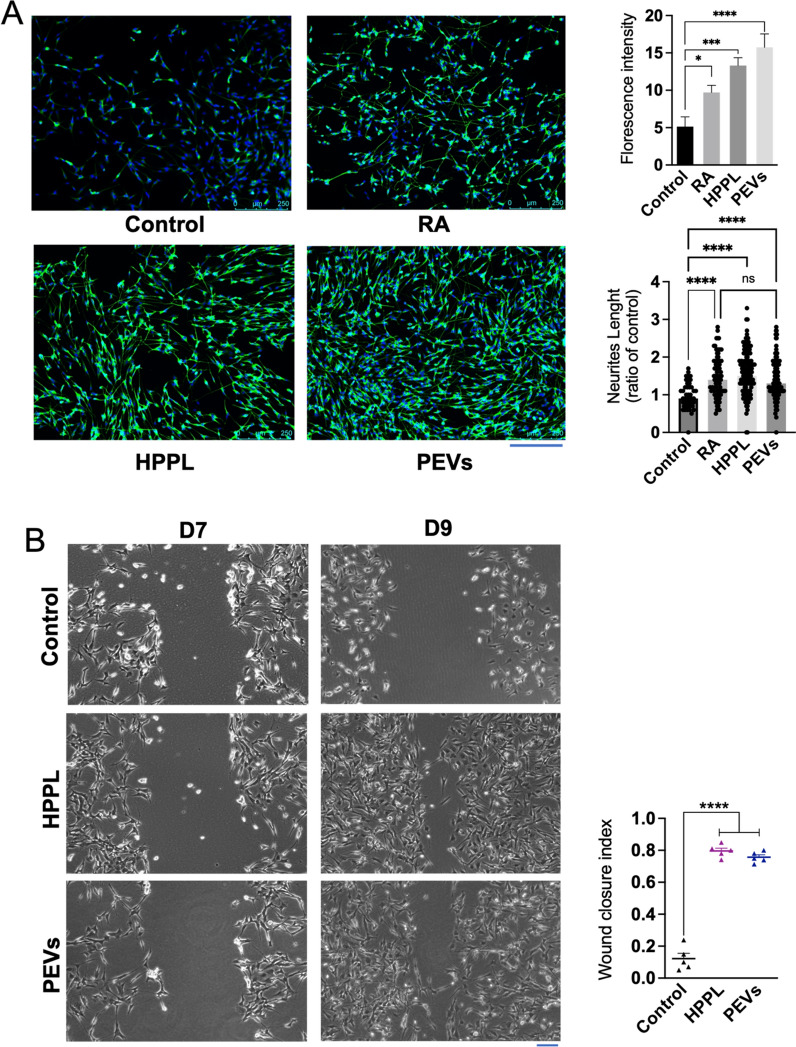
Functional activity of PEVs to promote neuronal growth in vitro on SH-SY5Y neuroblastoma cell line. **A** Capacity of PEVs to stimulate the neuronal maturation of SH-SY5Y cells. Cells were immuno-stained with β-III tubulin and counterstained with DAPI. Images showing extension of SH-SY5Y neurites under the treatment of PEVs and HPPL. HPPL and RA were used as positive controls to stimulate cell differentiation. Scale bar = 250 μm. Quantitative measurement of the fluorescence intensity (right) showed the capacity of PEVs to induce SH-SY5Y neuronal maturation. N = 3, *p < 0.05; ***p < 0.001 compared to the untreated negative control. From the captured images of β-III tubulin fluorescence, the length of each extension was measured individually to estimate total neurite outgrowth. The ratio of neurite length in treated cells to that in untreated cells was then calculated. N = 3, significant difference (ns), ****p < 0.001 compared to the untreated negative control. **B** Neuro-restoration effect of PEVs on the differentiated SH-5YSY. A scratch assay was performed using differentiated SH-SY5Y cells. Cells without any treatment (negative control), HPPL (positive control), and PEVs were used. The neuro-restoration effect was monitored by microscopy two days after the treatments on Day 9 (D9). Scale bar = 100 μm. The results are expressed as a wound-healing index, determined by the formula: (initial wound area − final wound area)/initial wound area). N = 3, ****p < 0.001 compared to untreated negative control. RA: Retinoic acid; PEVs: Platelet-extracellular vesicles; HPPL: heat-treated platelet pellet lysate

To evaluate the potential of PEVs in promoting neuronal differentiation in SH-SY5Y cells, an experiment was conducted in the absence of RA, a typical neuronal differentiation agent for this cell line. Initially, undifferentiated SH-SY5Y cells were cultured. On the second day, the culture medium was changed to DMEM without FBS. Subsequently, the cells were treated with PEVs, HPPL, or RA (the last two are used as positive controls). We assessed the differentiation marker β-III tubulin. Notably, cells treated with PEVs exhibited significantly higher relative fluorescence intensities of β-III tubulin (Fig. 5A) compared to the negative control (p < 0.01). Similar effects were observed in cells treated with HPPL (p < 0.01) and RA (p < 0.05). Additionally, the neurite outgrowth quantification showed that the neurite lengths in cells treated with HPPL and PEVs were significantly greater than in the control (untreated) group (p < 0.001), with no significant difference compared to the RA treatment. These data collectively demonstrate that PEVs effectively promoted SH-SY5Y differentiation, as found with HPPL positive control.

We further evaluated the neurorestorative capacity of PEVs by conducting a scratch assay on differentiated SH-SY5Y neuroblastoma cells. To initiate the experiment, SH-SY5Y cells were cultured and subjected to RA treatment for differentiation. A monolayer of these cells was then scratched using 100 μL pipette tips, followed by treatment with PEVs. The scratch area was monitored and imaged under a microscope for a period of two days (Fig. 5B). Results indicated that PEVs at the concentration used, effectively promotes neuronal growth, leading to closure of the scratched area, as what achieved by HPPL positive control. This contrasted with the control group, where no PEVs were applied, and the scratched area remained open. At the same time, the cell number also increased following treatment with PEVs and HPPL during neurorestoration. This increase occurred despite the absence of FBS in the differentiation medium, likely due to the growth factors present in PEVs and HPPL being sufficient to stimulate cellular growth and counteract the differentiation-induced inhibition of cell proliferation in this scratch assay.

### PEVs do not activate microglia BV-2 cells and reduce their activation by LPS

**Fig. 6:**
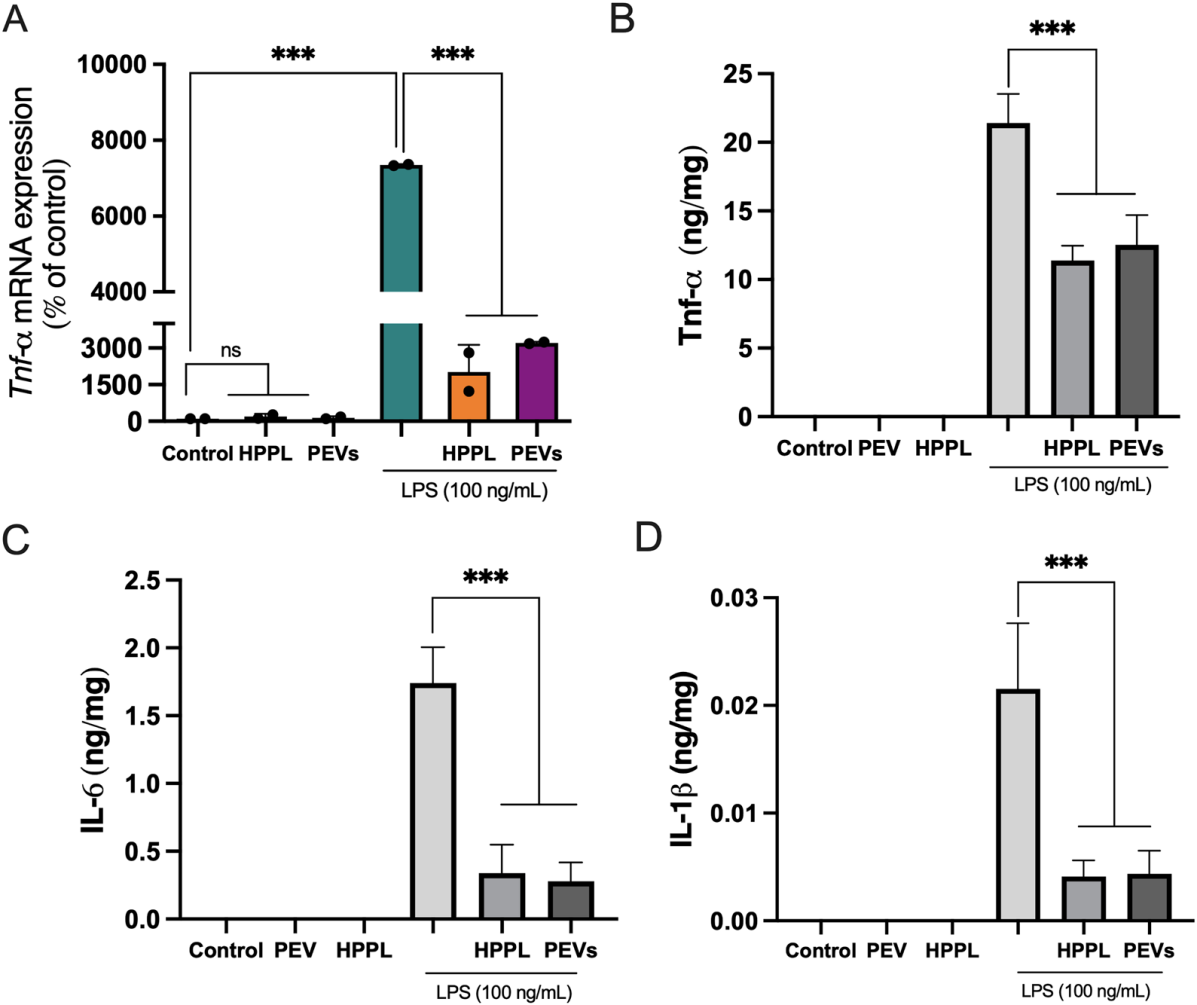
Anti-inflammatory activity of PEVs on activated BV-2 microglia cells. LPS was added to induce BV-2 cell activation 1 h before PEVs and HPPL (used as non-inflammatory control) treatments. The *Tnf-α* level was measured from RNA isolated from the cell lysate (pooled N = 2). TNF-α, IL-6 and IL-1β protein expression were quantified in the cell supernatant by ELISA (N = 3). The results showed that *Tnf-α* gene expression, TNF-α, IL-6, and IL-1β proteins were increased in BV-2 microglia cells exposed to 100 ng/ml LPS treatment, and a significantly lower expression was observed after 24 h of PEVs or HPPL treatments. ***p < 0.001 as compared to the control with LPS. ns: no significant difference; LPS: *Escherichia coli* lipopolysaccharide; PEVs: Platelet-extracellular vesicles; HPPL: heat-treated platelet pellet lysate

An in vitro study was performed using BV-2 cells to evaluate the impact of PEVs on microglial activation. BV-2 cells were cultured and subsequently treated with PEVs and HPPL, whereas untreated cells were used as a negative control. These treatments were assessed without and with LPS stimulation. We evaluated the pro-inflammatory cytokine Tnf-α, both at the gene and protein expression levels, as it is a major cytokine released by microglial cells upon LPS activation. Additionally, we examined other inflammatory markers, including IL-6 and IL-1β. Our results indicated that, under normal conditions without LPS stimulation, PEVs did not significantly induce Tnf-α gene expression, and there was undetectable expression of TNF-α, IL-6 and IL-1β proteins (Fig. 6). Similarly, a very low level of *Tnf-α* was detected by qPCR when the cells were exposed to HPPL control. In cells treated with LPS, the presence of PEVs or HPPL resulted in a significant reduction in the release of *Tnf-α* gene expression, as well as TNF-α, IL-6, and IL-1β protein expression compared to LPS treatment alone (p < 0.001).

### PEVs diffusion in the mice brain after i.n administration

This experiment aimed to assess the capacity of intranasally administered PEVs, using HPPL and PBS as controls, to diffuse within 7 h into the brains of mice. PBS, PEVs and HPPL samples were labeled using Alexa Fluor 568, and the free fluorescent dye was removed according to the supplier’s instructions. Compared to the PBS control, fluorescence slide scanner microscopic observation of brain slices revealed obvious red fluorescence diffusion within the brains of mice receiving the labeled PEVs and HPPL (Figure S4). As expected, fluorescent spots were absent in the PBS-labeled Alexa Fluor control group. The red fluorescence of labeled PEVs was evenly distributed throughout the brain, reaching key regions such as the cortex, hippocampus, thalamus, and striatum. This observation confirmed the penetration and widespread distribution of PEVs in the brain following intranasal administration, supporting our further exploration in subsequent in vivo models.

### PEVs exert anti-inflammatory activity in the in vivo model of CCI-TBI

**Fig. 7:**
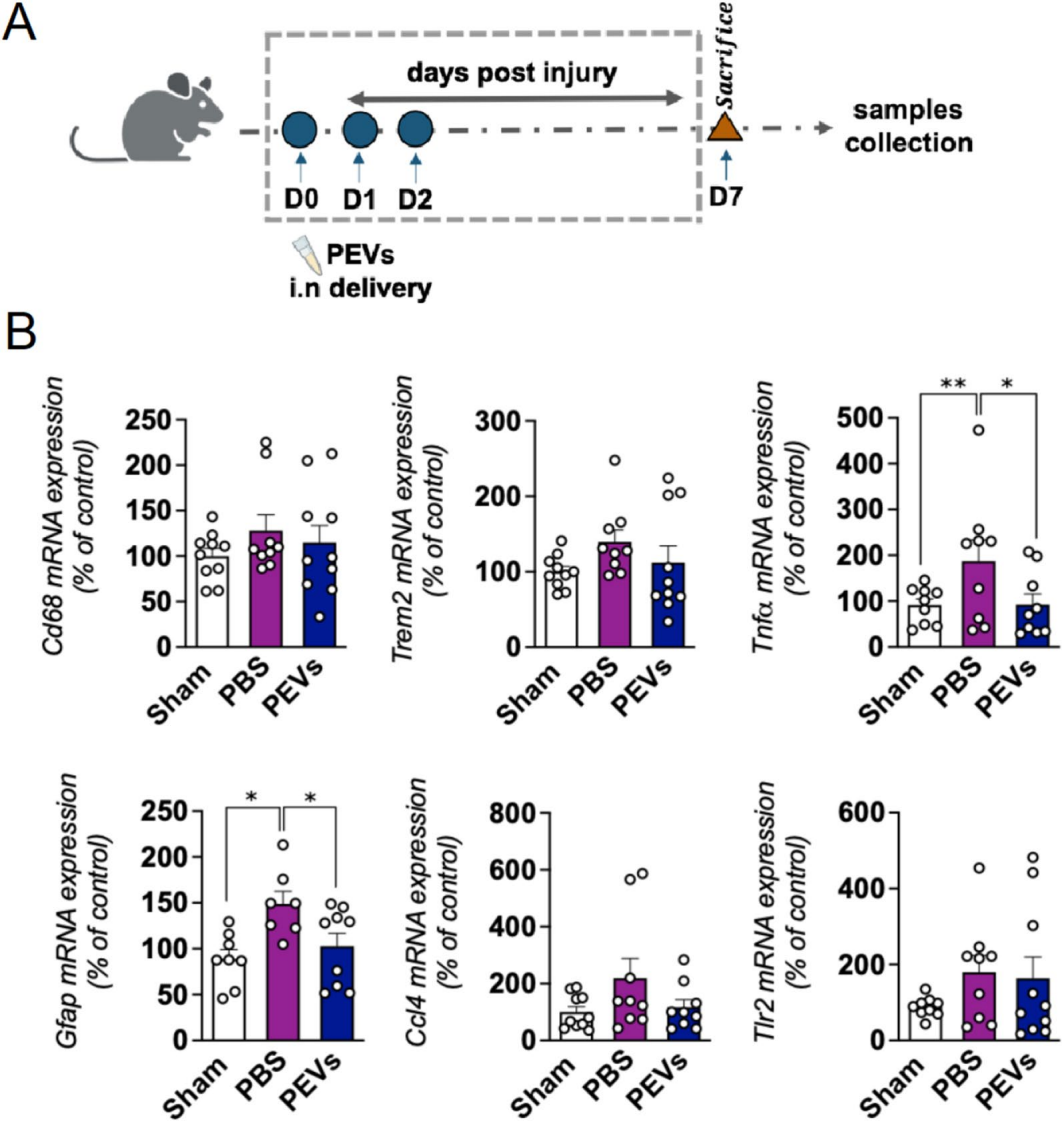
Anti-inflammatory activity of PEVs in CCI in vivo model of TBI. **A** Diagram illustrating the CCI-TBI mice to evaluate the anti-inflammatory effect of PEVs treatment. Mild CCI-TBI was applied on the left hemisphere of the mice brain followed by PEVs treatment administered intranasally (concentration 60 µL or 1.2 × 10^8^ on 3 consecutive days), and PBS was used as a control. On day 7, mice were sacrificed and the cortex injury part was collected for further gene expression analysis. **B** Effect of PEVs treatment on the expressions of inflammatory markers post injury. Changes in cytokines and glial markers expression in the cortex at day 7 post-injury by CCI. The inflammatory markers such as *Cd68*, *Trem-2*, *Tnf-α*, *Gfap*, *Ccl4*, and *Tlr2* were assessed. The results showed the upregulation of *Tnf-α*, *Cd68*, *Gfap*, and *Trem2* induced by the cortical impact and a significant suppression of *Gfap* and *Tnf-α* expression following PEVs treatment. Data were presented as means ± SEM (n = 7–10 in each group). *p < 0.05, **p < 0.01. CCI: Controlled cortical impact; TBI: Traumatic brain injury; PBS: Phosphate-buffered saline; PEVs: Platelet-extracellular vesicles

Following our in vitro studies, we used a mild CCI-TBI mouse model to examine the in vivo anti-inflammatory properties of intranasally delivered PEVs (Fig. 7A). The CCI was applied to the right hemisphere of the mouse brain. No adverse events were detected in both the PBS and experimental groups. One-week post-injury, we collected samples from the injured cortex and conducted assessments to determine the levels of selected pro-inflammatory markers including *Gfap*, *Cd68*, *Trem2*, *Ccl4, Tlr2*, and *Tnf-*$\alpha$. The results, as presented in Fig. 7B, showed the expected upregulation of the evaluated genes, such as *Tnf-*$\alpha$*, Cd68, Gfap, Trem2* induced by the cortical impact. Interestingly, there was a significant suppression of *Gfap and Tnf-*$\alpha$ expression following PEVs treatment.

### PEVs protected the TH positive cells in SN and improved motor behavior in MPTP-intoxicated mice

**Fig. 8:**
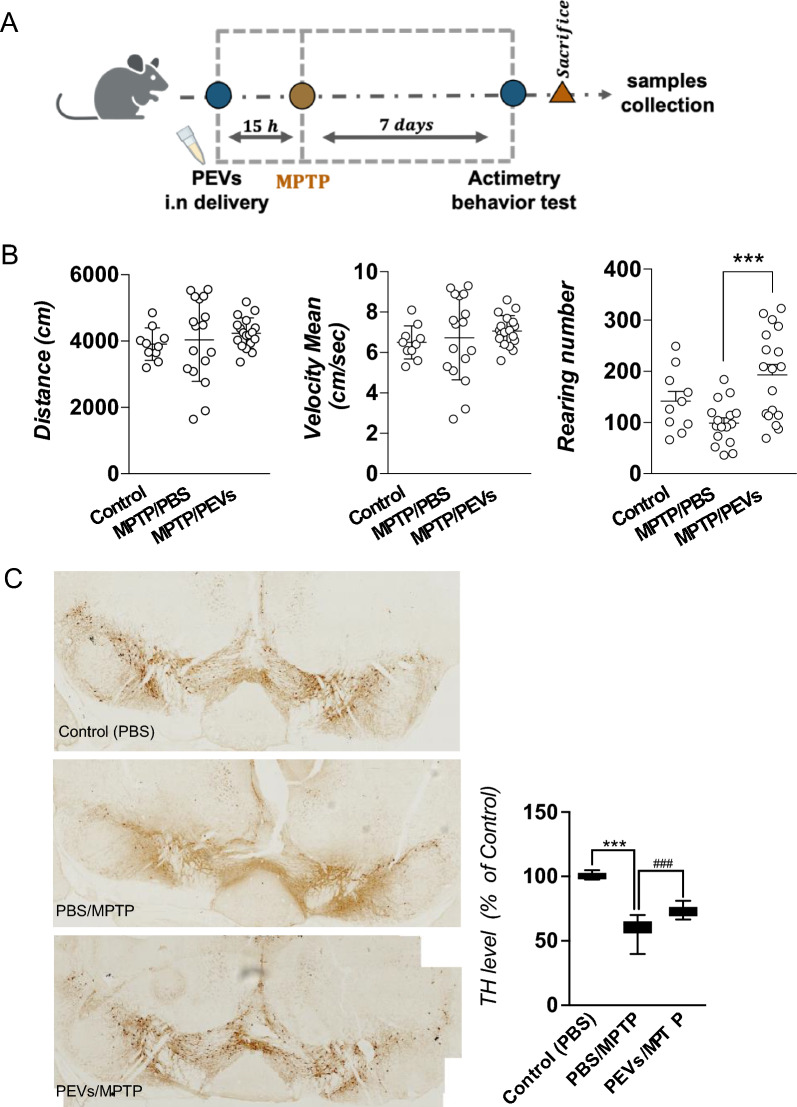
Neuroprotective effect of PEVs in MPTP mice model of PD. **A** Diagram illustrating MPTP mice to evaluate the neuroprotective effect of PEVs treatment. Approximately 15 h prior to the induction of MPTP intoxication (acute dose, 20 mg/kg MPTP injected intraperitoneally), intranasal (i.n) delivery of PEVs was administered at a dose of 4 × 10^10^. Some mice received saline as a control (sham group). On the seventh day, the open-field behaviour test was performed. After, the mice were sacrificed, and their brains were collected for IHC analysis. **B** Impact of the PEVs treatment on the behaviour of MPTP mice. Animals were left in the open field actimetry box and allowed to explore freely for 10 min. The test was done on day 7. Parameters related to their locomotor function were recorded. PEVs given by i.n. delivery prior to MPTP intoxication improved the rearing number compared to the control MPTP group (MPTP/PBS). **C** Effect of PEVs treatment on the TH positive cells in SN of MPTP mice. On the left, a representative images of TH area counted in the SN area compared between the control/PBS group and MPTP groups either receiving PBS (MPTP/PBS) or PEVs (MPTP/PEVs). Total TH number in the subsequent area of SN (7 sections collected between bregma – 2.92 mm and – 3.88 mm). The TH counts in each section were summed for each animal. The treatment by PEVs was able to protect TH expression. Data presented as means ± SD, n = 7 in the sham group (PBS only), and at least n = 17 in the MPTP groups. ***p < 0.001 compared to PBS group, ^###^p < 0.001 compared to MPTP/PBS group. MPTP: 1-methyl-4-phenyl-1,2,3,6-tetrahydropyridine; PBS: Phosphate-buffered saline; PEVs: Platelet-extracellular vesicles; SN: Substantia nigra; TH: Tyrosine hydroxylase

Male C57BL/6 mice received i.n. administration of PEVs 15 h before the first intoxication by MPTP, a neurotoxin used to simulate PD symptoms (Fig. 8A). No adverse events were detected in both the PBS and experimental groups. To evaluate the impact of PEVs on locomotor function in MPTP-treated mice, we conducted an open-field behavior test using an actimetry device. Mice were allowed to roam freely, and their movements were recorded for 10 min. Tests were performed on Day 7 post MPTP/PEVs treatment. We observed high variability in recorded parameters on MPTP/PBS group such as in the total distance and velocity indicating the impact of MPTP treatment in the disruption of normal mouse behavior, causing erratic movement patterns (Fig. 8B). This observation suggests that the deleterious action of MPTP remained moderate in this experiment. Notably, in the PEVs-treated group, the variance in the measured parameters was diminished, aligning more closely with the characteristics of the sham group. Importantly, the number of rearing events, which are known to be the most sensitive parameter adversely affected by MPTP, was significantly increased (p < 0.01) in the group receiving MPTP and PEVs compared to the group receiving MPTP and PBS. Following TH staining, we counted the total number of TH^+^ cells in the SN. This was done bilaterally in seven brain sections ranging between bregma − 2.92 mm and − 3.88 mm, as routinely done in our laboratory [34]. For each animal, the neuron counts from each section were summed to obtain a total count. A significant reduction of TH-positive cells was observed in the mice intoxicated with MPTP as expected (p < 0.001). This reduction confirmed the effect of MPTP. Interestingly, the i.n. administration of PEVs prior to MPTP intoxication significantly prevented the death of dopaminergic neurons in SN from MPTP-induced damage (p < 0.001), as illustrated in Fig. 8C.

## Discussion

Our study successfully demonstrates for the first time—to our knowledge—the isolation of functional PEVs from the supernatant of pooled, anticoagulated, clinical-grade PC and their evaluation in models of neurological disorders. Both our cellular and animal experiments revealed multiple roles of PEVs in neuroprotection. Specifically, PEVs aided the repair of SH-SY5Y neuroblastoma cells by facilitating neuronal reconnection and enhanced the maturation of these cells, as indicated by the strong expression of β-III tubulin. Prior studies have shown that growth factors such as PDGF, BDNF, and IGF-1 are involved in the survival and differentiation of SH-SY5Y cells [35–37]. Consistent with our current findings, our group observed similar benefits using the HPPL platelet lysate enriched with various growth factors, such as PDGF and BDNF [38]. The neuroprotective effects observed in LUHMES cells are also attributed to a mix of functional growth factors, chemokines, and antioxidants, including glutathione peroxidases (GPX, including GPX-4) and SOD1. These components collectively work to reduce lipid peroxidation and shield cells from ferroptosis following erastin exposure [37, 39, 40]. PEVs have also been shown to have anti-inflammatory and restorative effects, particularly by inhibiting Tnf-α, a key inflammatory cytokine, at both gene and protein levels, as well the protein level of IL-6, IL-1β released by BV-2 microglia after LPS activation [41, 42]. Importantly, PEVs did not trigger activation of BV-2 microglia in the absence of LPS stimulation, which is important to support various applications in neurodegenerative disorders where neuroinflammation is detrimental [43, 44].

Intranasal administration, a delivery method that bypasses the BBB [45], has been shown to be valuable for the administration of nanoscale EVs and their therapeutic load to the brain [46, 47]. Our fluorescent imaging confirms that labeled PEVs penetrate and diffuse into the brain, as observed with HPPL used as a control. This is consistent with our recent study, which demonstrated the diffusion of intranasal HPPL, also labeled with Alexa Fluor, into the brains of mice, including the hippocampus, within 7 h of administration [33]. This same study also showed the capacity of intranasal HPPL to modify the hippocampal proteome, as evidenced by differentially expressed proteins (DEPs), further confirming the efficacy of the intranasal route in mice. In our previous studies, intranasal HPPL protected TH expression in the substantia nigra and provided neuroprotective effects in the MPTP mouse model of PD [9]. It also enhanced memory functions, alleviated cortical neuroinflammation, and reduced oxidative stress in a TBI model [7]. Together, our findings support the potential for intranasal delivery of PEVs to be as effective as that of HPPL in reaching various brain areas, including the cortex and the substantia nigra, and in exerting neuroprotective effects.

Our data demonstrated how PEVs modulate inflammatory responses following TBI in mice. PEVs and the HPPL positive control were administered intranasally over three consecutive days, and their anti-inflammatory effects in ipsilateral cortical tissues were evaluated 7 days post-injury. Both treatments were given at a dose of 60 μL/day, totaling 180 μL over 3 days. This dosing selection was based on our previous in vivo studies [7, 8] and taking into consideration, as a reference, the number of PEVs present in HPPL [22, 31]. In addition, our proteomics analysis identified 1117 and 652 proteins with a protein-level FDR of 1% in HPPL and PEVs, respectively (Figure S5). Notably, the Venn diagram showed that 595 of these proteins, representing 90% of the PEV proteins, were also found in HPPL. This highlights a significant similarity in their protein profiles. The anti-inflammatory effect of PEVs was demonstrated by reduced levels of *Tnf-α, Cd68, Gfap, and Trem2* in the brains of mice treated with PEVs after CCI. This finding is consistent with previous results obtained using the HPPL preparations in the same model [7, 8]. In the present study, our primary focus was on analyzing the anti-inflammatory activity of PEVs in the injured brain of CCI-TBI. Following an injury, an immediate cascade of inflammatory responses occurs, and modulating this phase is crucial to prevent further damage. Thus, we chose to test the impact of PEVs on the inflammatory response in this model. However, we acknowledge that other aspects of brain pathology and functional outcomes need to be evaluated. This has been addressed in our previous studies with HPPL in CCI, showing positive outcomes in synaptic protection and cognitive activity following the modulation of inflammation [7]. The similar anti-inflammatory impact of PEVs and HPPL, along with the similarity in their proteomic profiles, supports the likelihood of comparable functional effects, but further studies are needed to confirm this assumption.

Additionally, in the standard model of PD induced by acute MPTP intoxication, i.n. delivery of PEVs protected TH-positive neurons, showing a preservative effect on dopaminergic neurons in adult mice. This protection was linked to a marked improvement in rearing behavior, which is a sensitive indicator of alterations in dopamine neurotransmission in this model. One limitation of this experiment is the high variation observed in the MPTP/PBS group, which does not result in a statistically significant difference compared to the sham control in recorded parameters such as total distance and velocity. However, the increased variability in these parameters within the MPTP/PBS group suggests an effect of the MPTP treatment. The inconsistent movement patterns observed in mice indicate the impact of MPTP, highlighting its disruptive effect on normal behavior. Our results show that PEVs treatment reduced this variability, indicating a mitigating effect on the disruptive impacts of MPTP. Additionally, the number of rearing events, which are known to be most adversely affected by MPTP, was significantly increased. This underscores the likely efficacy of PEVs treatment in ameliorating the behavioral impacts associated with MPTP treatment. The neuroprotective, neuroregenerative, and anti-inflammatory effects of PEVs demonstrated both in vitro and in vivo in our studies is likely mediated by a range of growth factors, antioxidants, and cytokines present within the PEVs. A synergy of various platelet growth factors has been shown to boost the proliferation and differentiation of neuronal stem cells. Moreover, blocking individual growth factors did not completely negate these effects [48, 49], which supports the rationale for the multifaceted biotherapeutic approach in scientific and clinical settings using PEVs [1].

The PEVs we used were derived from a standardized source material, and purified through a well-described, reproducible and reasonably scalable process involving sequential centrifugation. We conducted a thorough characterization of the PEVs to establish a foundation for standardization and future translational applications. These PEVs had a main population size ranging from 70 to 350 nm, predominantly around 200 nm, consistent with our previous study [30]. The minor population of events with a mean size of 20 nm detected by DLS could correspond to either residual plasma proteins co-purified with the PEVs during ultracentrifugation, or to a loose protein corona forming on the PEVs [50]. For any further research or translational developments, this 20-nm population could likely be removed by size exclusion chromatography using commercially available resins with a cut-off value above 35 or 70 nm [22, 51]. The purified PEVs exhibited the expected characteristics of lipid bilayer vesicles, including morphology, size, markers, and molecular protein composition, aligning with current guidelines on EVs [52, 53]. These PEVs also express membrane markers typical of platelets and EVs, as shown by EV antibody membrane array and WB analysis, and corroborated by other studies [22, 54–56]. Our EV antibody membrane array and WB study did not include the demonstration of the absence of negative markers. However, we performed a proteomic analysis following the guidelines from MISEV2018 and MISEV2023 for EV protein characterization and classification, as outlined in Supplementary Table 3. In category 3, the presence of certain proteins, such as those related to lipoproteins, can serve as negative EV markers to assess purity. Our analysis showed that apolipoproteins, such as APOB100, as well as albumin (ALB), and proteins related to nucleic acid aggregates, such as Tamm-Horsfall protein (Uromodulin/UMOD), were not detected in our PEV preparation. Notably, at least 69 identified proteins in our PEVs are listed among the top 100 well-known EV proteins in Vesiclepedia and Exocarta EV protein databases, confirming the richness in EVs in our sample. Additionally, these PEVs display low pro-coagulant activity in vitro, comparable to a platelet lysate designed for brain applications [9, 31], a critical factor for brain administration. In our laboratory tests, the PEVs enhanced cell growth and contributed to the repair, differentiation, and maturation of neuroblastoma SH-SY5Y cells. They also provided protection to LUHMES dopaminergic neurons from erastin-induced ferroptosis. Cell morphology and viability were used as primary criteria to assess the role of PEVs in protection against erastin. Our team is currently investigating various ferroptosis-specific pathways in more detail to elucidate the mechanisms underlying PEVs' protective activity against erastin and RSL-3, both of which are known ferroptosis inducers. This is relevant as proteomic analysis has revealed the presence of GPX-1, GPX-3, GPX-4, and a mix of antioxidative proteins such as glutathione S-transferase, catalase, peroxiredoxin-6, and the mitochondrial enzymes SOD1 and SOD2. Together, these proteins may play a significant role in restoring the brain's redox balance and reducing ROS caused by ferroptosis [57]. Also, the PEVs demonstrated anti-inflammatory and reparative effects in BV-2 cells triggered by LPS. The i.n. administration of PEVs mitigated inflammation in a CCI-induced TBI model. Furthermore, they showed neuroprotective effects of dopaminergic neurons in the SN and improved motor functions in a mouse model of PD induced by MPTP neurotoxin. The isolation procedure does not allow us to ascertain whether the functional activity is associated with small or large PEVs, or both. However, ultracentrifugation at a gravitational force of 25,000×*g* for 90 min is more inclined to pelletize the largest particles. Recovery of the smallest EVs, usually referred to as "exosomes" with typical sizes ranging from 20 to 80 nm, requires higher g force and longer ultracentrifugation durations (at least 100,000×*g* for 2 h). Additionally, the main DLS peak of ~ 200 nm suggests that the majority of the isolated PEVs are more likely to correspond to larger platelet EVs. This assumption is supported by NTA and TRPS, which have confirmed the size distribution of PEVs, as well as cryoEM, which showed vesicle sizes of ~ 50–300 nm, with most being around 200 nm.

Isolating PEVs from the supernatant of PCs offers multiple translational benefits for treatment of brain disorders using domestic blood supply. First, the clinical-grade PCs were sourced from the Taiwan Blood Services Foundation, a certified blood collection facility. These PCs were obtained using standardized procedures from healthy, volunteer non-remunerated donors. Furthermore, each donation underwent testing for key viral markers, in compliance with the safety level necessary for clinical applications. The World Health Organization has indeed listed PCs as essential medicines for adults and children, highlighting the need for standardized quality, safety, and consistent national supply on a global scale including in low- and middle-income countries [58]. Second, isolation from clinical-grade PCs collected with citrate anticoagulant minimizes platelet activation and generation of pro-coagulant PEVs, as found here. In contrast, we have shown that PEVs generated by thrombin activation of isolated platelets exhibit strong PS expression, promote immediate aggregation of THP-1 monocytic cells [59] and neutrophil extracellular trap formation [60]. Specialized coagulation assays, such as the MP-PS or STA-PPL we used in our study, are valuable for process development to monitor PEVs safety for CNS therapies where clot formation could be detrimental [61]. Third, pooling PEVs from several donations is feasible, minimizing variations between individual donors to achieve a more uniform product, as proven for cell therapies [13, 62]. Such pooling should nevertheless consider, based on risk assessment, the need for additional measures of viral safety such as pathogen reduction treatment of the PCs. Fourth, the isolation method from the PC supernatant enabled us to recover as a side-fraction, the concentrated platelets for further research or use in neuroregenerative medicine [1, 61], thus reducing waste of precious blood resources. We also utilized "outdated" PCs—those not used within 5 days of collection and deemed unsuitable for transfusion—ensuring that this development does not affect hospital needs. Our research confirms that these outdated PCs are a viable and efficient source for harvesting functional PEVs. This approach is consistent with the successful use of pooled platelet lysates from outdated PCs for the ex vivo expansion of therapeutic human cells [61, 62]. These practical considerations are vital to facilitate the clinical use of these PEVs.

In our study, we used a combination of sequential centrifugation and ultracentrifugation to separate PEVs from the supernatant of PCs. The isolation procedure was performed in a closed system, utilizing sterile apparatus and solutions, to ensure aseptic processing and maintain sterility throughout. This method also circumvents the requirement for bacterial filtration using 0.2 μm filters, which could potentially reduce the yield of PEVs, a concern particularly relevant when dealing with small volume samples. However, differential centrifugation procedures remain somewhat limited in their scalability [63, 64]. We could explore alternative scalable techniques for isolating PEVs, such as size-exclusion chromatography, which has been well documented in several studies [22, 51, 65] and can expand product availability.

Importantly, our study explored the protein composition of the PEVs to identify functional components for neuroprotection. Until recently, characterization of the trophic factor content of platelet biomaterials relied on ELISA determination of singular bioactive molecules, such as BDNF, PDGF, EGF, VEGF, or PF4. These factors are abundant in platelets, especially their α-granules, making their quantifiable presence in our PEVs anticipated [9, 10, 14, 66]. They are known promoter of neuronal growth and repair [1, 67–70]. Among the factors we analyzed by ELISA, BDNF protects neuronal damages from TBI [71] and supports the survival and function of dopaminergic neurons [72], which are crucially impacted in PD. Similarly, PDGF enhances the functional recovery from TBI by inhibiting the endothelium reticulum stress and autophagy and by decreasing the expression of various pyroptosis-related proteins, including NLRP3 (nucleoside-binding domaine leucine-rich-containing family, pyrin domain-containing-3) inflammasome and pro-Caspase1, among others [73]. The high content of PF4, over 300 ng/mL, deserves further investigation of its functional contribution to our PEVs as very recent studies have highlighted the role of this chemokine in improving cognition in several mouse models [17, 74].

Although ELISA is a sensitive quantitative technique it has limited applicability in providing an extended profiling of complex biological mixtures. Therefore, one pivotal aspect of our study lies in the proteomic analysis which we conducted. Proteomic offered an in-depth holistic profile of the complex biochemistry of PEVs, allowing a multifaceted understanding of the biological functions. The data revealed that the PEVs contain a rich assortment of neurotrophic factors, anti-inflammatory proteins, and antioxidants [e.g. hepatocyte growth factor (HGF), transforming growth factor (TGF)-β1, IGF, catalase, glutathione S-transferase, GPXs, SOD 1,2, glutamate-rich protein and CAMP-dependent protein kinase related proteins]. Altogether, this combination may mitigate neuronal impairments, and provide a rationale for the anti-inflammatory and neuroprotective effects observed in both TBI and PD models. For instance, SOD can preserve cellular redox balance and combat harmful reactive oxygen species generated in TBI [75, 76]. CAMP-dependent protein kinase, a major component identified in PEVs, plays a crucial role in multiple physiological functions. This includes neuronal formation and survival as highlighted in various studies [45, 77]. Our FunRich analysis also underscored the importance of PEVs in cellular communication, signal transduction, and cell growth and maintenance. A noticeable finding was the detection of CCL5 (RANTES), a chemokine with recently discovered neurotrophic effects. I.n. administration of CCL5 has been shown to counteract ROS and inflammatory chemokines, leading to cognitive and memory improvements after TBI, likely through GPX-1 antioxidant stimulation [16]. Furthermore, CCL5 was found to play a beneficial function in synaptic protein expression, neuronal connectivity and cognitive function [15]. Additionally, the presence of GTPase in our analysis implies its involvement in vital cellular processes, such as growth and differentiation, as noted in previous studies [78]. Other molecular functions we identified, including cytoskeletal binding, protease inhibition, catalytic activity, transporter activity, protein-tyrosine kinase activity, and protein binding may facilitate signaling and interactions between EVs and cells, as suggested before [27].

Furthermore, the proteomic analysis of PEVs presents several practical benefits, particularly for monitoring batch consistency. By quantifying the concentrations of various bioactive molecules within PEVs, it could be possible to identify the most prevalent markers for in vivo dosing, potentially reducing the risk of side effects. Moreover, this analysis enables comparisons between different PEV populations and enhances our understanding of the functional distinctions between PEVs and EVs from other cellular origins, such MSCs. This could be instrumental in addressing current gaps in our knowledge. Thus, a thorough proteomics study of PEVs could reveal new therapeutic agents for neurodegenerative diseases. It might also refine strategies for engineering PEVs by enriching them with specific growth factors and antioxidants, potentially augmenting their therapeutic efficacy for treating neurological conditions [79].

One limitation of our study is that we did not analyze the miRNA content within the PEVs. Including miRNA profiling, alongside proteomics, could significantly enhance our understanding of the functional impacts on neuronal cells and would be a valuable addition to future research. Another point to consider is the lack of investigation into the effects of photochemical pathogen reduction treatments on PCs, which are becoming more widely licensed across various countries [80], on PEV functionality. This aspect is particularly crucial when PEVs are pooled from multiple donors, as it could have implications for clinical trials and commercial manufacturing. However, in previous studies, we have found that a photochemical pathogen inactivation treatment using a combined psoralen and UVA exposure does not affect the neuroprotection by HPPL [81]. A limitation of our TBI study is the focus on anti-inflammatory markers. While our findings contribute valuable insights into the anti-inflammatory effects of PEVs consistent with previous investigations using platelet lysate [7, 8], this choice might have overlooked other crucial aspects of TBI pathology, such as neuroregeneration or synaptic plasticity. Also, in the MPTP model of PD, we focused on assessing rearing behavior as a primary measure of motor function. This choice represents a limitation due to the exclusion of other behavioral assessments relevant to PD pathology, such as bradykinesia, balance, and coordination tests. Therefore, future research using PEVs isolated from pathogen-reduced PCs for clinical translation could benefit from a more comprehensive analysis of TBI outcomes, including a broader range of neuroprotective and regenerative markers, to fully elucidate the therapeutic potential of PEVs in this context, and might consider incorporating a wider array of behavioral tests to capture a more holistic view of motor function improvements in PD models. In addition, while our study provides proof of concept for the potential of intranasal PEVs in TBI and PD, future preclinical studies must consider the severity of these diseases to define the optimal volume and dosing of administration. This remains a crucial step that needs to be carefully assessed to maximize therapeutic efficacy.

## Conclusions

Our findings on the therapeutic potential of PEVs in treating brain disorders have significant implications for the broader fields of regenerative medicine and neurology [61]. These PEVs prepared from clinical-grade PC supernatants from healthy donors have a rich assortment of neurotrophic factors, antioxidants, and anti-inflammatory proteins. The versatility of PEVs in addressing various pathological processes, such as inflammation, ferroptosis, and neuronal damage, may offer a multifaceted approach to various CNS disorders. Our findings also serve as a proof-of-concept for the therapeutic value of intranasal PEVs in the brain, though further pre-clinical research is needed to determine to optimize dosage for specific brain applications. Intracranial, topical, or intracerebroventricular administration of platelet materials can also be explored as done in preclinical studies in cerebral ischemia [48] and ALS mouse models [82]; they are more invasive and complex to implement for animal studies and clinical use, but they may allow a better control of the dose reaching specific brain areas. While the anti-inflammatory and neuroprotective effects of PEVs are clear in our two models of brain pathology, comprehensive evaluation in long-term pre-clinical models is still required. Scaling up PEV production for clinical applications will present some challenges [64], yet recent developments suggest it is feasible, as demonstrated in placebo-controlled clinical trials of other PEVs for wound healing [83]. The process of purifying our specific population of PEVs from the same PCs that can be used for platelets isolation could optimize the utilization of human platelet donations and reduce treatment costs—a significant advantage globally- making this therapeutic approach potentially implementable also in low- and middle-income countries using domestic blood resources.

## Supplementary Information

Supplementary Material 1. Table S1. List of primers used in this study

Supplementary Material 2. Table S2. PEVs proteins list

Supplementary Material 3. Table S3. Protein profiling and characterization of PEVs: a comprehensive analysis

Supplementary Material 4. Figure S1. Representative images of neurite outgrowth quantification

Supplementary Material 5. Figure S2. Western blot analysis of PEV markers

Supplementary Material 6. Figure S3. Procoagulant assays

Supplementary Material 7. Figure S4. PEVs diffusion in the mice brain

Supplementary Material 8. Figure S5. Venn Diagram of common proteins present in both PEVs and HPPL

## Data Availability

Data and materials are available from the corresponding authors upon reasonable request.

## Abbreviations

BBB: Blood–brain barrier
BCA: Bicinchoninic acid
BDNF: Brain-derived neurotrophic factor
BSA: Bovine serum albumin
CCI: Controlled cortical impact
CCK-8: Cell counting kit-8
CNS: Central nervous system
DLS: Dynamic light scattering
DMEM: Dulbecco’s modified Eagle’s medium
EGF: Epidermal growth factor
ELISA: Enzyme-linked immunosorbent assay
EVs: Extracellular vesicles
FBS: Fetal bovine serum
FDR: False discovery rate
HPL: Human platelet lysate
HPPL: Heat-treated platelet pellet lysate
IGF: Insulin-like growth factor
IHC: Immunohistochemistry
LC–MS/MS: Liquid chromatography-tandem mass spectrometry
LPS: Lipopolysaccharide
LUHMES: Lund human mesencephalic
MP: Microparticle
MPTP: 1-Methyl-4-phenyl-1,2,3,6-tetrahydropyridine
NTA: Nanoparticle tracking analysis
PBS: Phosphate buffer saline
PC: Platelet concentrate
PD: Parkinson’s disease
PS: Phosphatidylserine
PDGF: Platelet-derived growth factor
PEVs: Platelet extracellular vesicles
PF4: Platelet factor-4
PFA: Paraformaldehyde
PLT: Platelet
PPL: Platelet pellet lysate
RA: Retinoic acid
SN: Substantia nigra
SOD: Superoxide dismutase
STA-PPL: STA-procoagulant-phospholipid assay
TBI: Traumatic brain injury
TH: Tyrosine hydroxylase
TRPS: Tunable resistive pulse sensing
VEGF: Vascular endothelial growth factor
