# Supplementary material for: Neuroprotective effects of intranasal extracellular vesicles from human platelet concentrates supernatants in traumatic brain injury and Parkinson’s disease models
Source: J Biomed Sci. 2024 Sep 5;31:87. doi: 10.1186/s12929-024-01072-z (PMC11375990; doi:10.1186/s12929-024-01072-z)
Supplement: Supplementary file 1 — Supplementary Material 1. Table S1. List of primers used in this study [file 12929_2024_1072_MOESM1_ESM.docx]

**Supplementary Table 1: list of the primers used in this study**

| **Name** | **Forward primer** | **Reverse primer** |
| --- | --- | --- |
| Cyclophilin | agcatacaggtcctggcatc | ttcaccttcccaaagaccac |
| CD68 | gacctacatcagagcccgagt | cgccatgaatgtccactg |
| TREM2 | cgagaggctgaggtcctg | tctccagcatcttggtcatcta |
| TNF-$\alpha$ | tgcctatgtctcagcctcttc | gaggccatttgggaacttct |
| GFAP | cgcgaacaggaagagcgcca | gtggcgggccatctcctcct |
| CCL4 | gccctctctctcctcttgct | gagggtcagagcccattg |
| TLR2 | ggggcttcacttctctgctt | agcatcctctgcgatttgacg |
