# Supplementary material for: Neuroprotective effects of intranasal extracellular vesicles from human platelet concentrates supernatants in traumatic brain injury and Parkinson’s disease models
Source: J Biomed Sci. 2024 Sep 5;31:87. doi: 10.1186/s12929-024-01072-z (PMC11375990; doi:10.1186/s12929-024-01072-z)
Supplement: Supplementary file 3 — Supplementary Material 3. Table S3. Protein profiling and characterization of PEVs: a comprehensive analysis [file 12929_2024_1072_MOESM3_ESM.docx]

| **Categories** | **1. Transmembrane or GPI-anchored proteins associated to plasma membrane and/or endosomes** | **2. Cytosolic proteins recovered in EVs** | **3. Major components of non-EV co-isolated structures** | **4. Transmembrane, lipid-bound and soluble proteins associated to other intracellular compartments than plasma membrane /endosomes** | **5. Secreted proteins recovered with EVs** |
| --- | --- | --- | --- | --- | --- |
| **Present in PEV** | **1a: non-tissue specific**  Tetraspanins (CD63, CD81); other multi-pass membrane proteins (CD47; GNA13, GNAQ, GNA12), MHC class I (HLA-A, HLA-B, HLA-C), Integrins ( ITGA2  ITGA2B, ITGB3, ITGB1), EMMPRIN (BSG); ADAM10  **1b: cell/tissue specific**  PECAM1 (endothelial cells), (immune cells), CD41 (ITGA2B) and CD42a (GP9) (platelets), | **2a**: **with lipid or membrane protein-binding ability**  ALIX (PDCD6IP), EHD1, EHD3; RHOA; annexins (ANXA1, ANXA3, ANXA5, ANXA7); HSPA4, HSPA1B, (HSPA8), and HSP84 (HSP90AB1)  **2b: promiscuous incorporation in EVs**  Heat shock protein HSP70 (HSPA1A), cytoskeleton: actin (ACTR1A, ACTC1, ACTB, ACTR3, ACTR2, ACTBL2), tubulin (TTUBB, TUBB1,  TUBB4A, TUBB4B, TUBA8, TUBA1C); enzymes (GAPDH) | **3a: lipoproteins**  Apolipoproteins A1/2 and B APOA1/2, APOB;  **3b: protein and protein/nucleic acid aggregates**  ribosomal proteins (RPLP2) | **4a: nucleus (-)**  **4b: mitochondria**  SOD1, SOD2  **4c: secretory pathway**  calnexin (CANX); Grp94 (HSP90B1); BIP (HSPA5),  **4d: others (autophagosomes, cytoskeleton…)**  Actinin1/4 (ACTN1/4), | **5a: Cytokines and growth factors**  CCL5, PF4, PF4IV1, catalase (CAT), GPX1, GPX3, GPX4, GRID1; TGM7, SH3BGRL, PRKACB, PRKAR1A, PRKAR1B, PRKAR2A, PRKAR2B, EGF receptors,  GRB2, HGFAC, IGF2, IGFALS, LTBP1, TGFB1/3, TGFB1I1, EFEMP1  **5b: adhesion and extracellular matrix proteins**  Fibronectin (FN1 galectin3-binding protein (LGALS3BP), CD5L; fetuin-A (AHSG) |
| **Not found in PEV** | **1a: non-tissue specific**  Transferrin receptor (TFR2); LAMP1/2; SDC; GPI-anchored 5ʹnucleotidase CD73 (NT5E), complement-binding proteins CD55 and CD59; sonic hedgehog (SHH) | **2a**: **with lipid or membrane protein-binding ability**  exomeres; ARF6; syntenin (SDCBP); microtubule-associated Tau (MAPT, neurons)  **2b: promiscuous incorporation in EVs (-)** | **3a: lipoproteins**  APOB100; albumin (ALB)  **3b: protein and protein/nucleic acid aggregates**  Tamm-Horsfall protein (Uromodulin/UMOD) | **4a: nucleus**  Histones (HIST1H**); Lamin A/C (LMNA)  **4b: mitochondria**  IMMT, cytochrome C (CYC1); TOMM20  **4c: secretory pathway**  Golgi apparatus, GM130 (GOLGA2)  **4d: others (autophagosomes, cytoskeleton…)**  ATG9A, cytokeratin 18 (KRT18) | **5a: Cytokines and growth factors**  interleukins (IL*)  **5b: adhesion and extracellular matrix proteins**  Collagen (COL**), MFGE8 |

**Supplementary Table 3. Protein profiling and characterization of PEVs: a comprehensive analysis (based on MISEV 2018/2023 categories)**
