# Supplementary material for: Neuroprotective effects of intranasal extracellular vesicles from human platelet concentrates supernatants in traumatic brain injury and Parkinson’s disease models
Source: J Biomed Sci. 2024 Sep 5;31:87. doi: 10.1186/s12929-024-01072-z (PMC11375990; doi:10.1186/s12929-024-01072-z)
Supplement: Supplementary file 4 — Supplementary Material 4. Figure S1. Representative images of neurite outgrowth quantification [file 12929_2024_1072_MOESM4_ESM.docx]

*
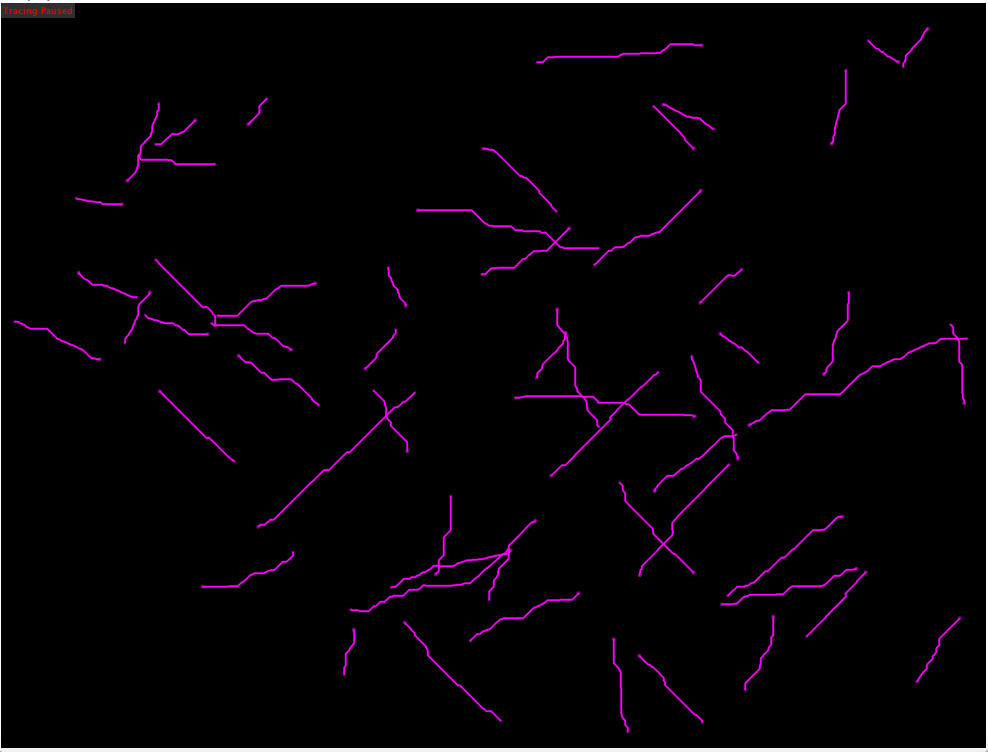

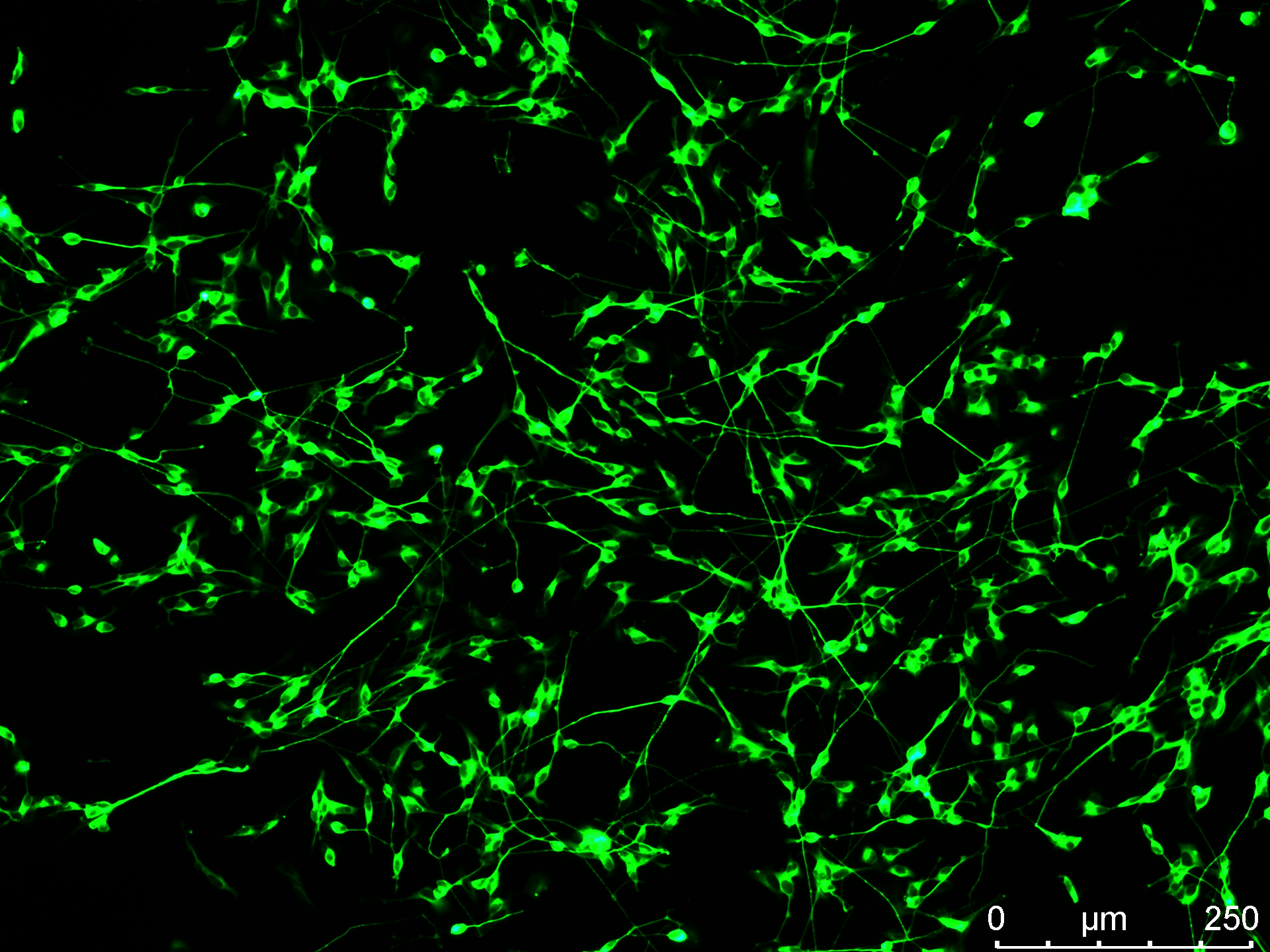
*

FIGURE S1. Representative images of neurite outgrowth quantified using the FIJI plugin Simple Neurite Tracer (SNT.V4.2.1), based on B-III tubulin fluorescence images of SH-5YSY cells.
