# Supplementary material for: Neuroprotective effects of intranasal extracellular vesicles from human platelet concentrates supernatants in traumatic brain injury and Parkinson’s disease models
Source: J Biomed Sci. 2024 Sep 5;31:87. doi: 10.1186/s12929-024-01072-z (PMC11375990; doi:10.1186/s12929-024-01072-z)
Supplement: Supplementary file 5 — Supplementary Material 5. Figure S2. Western blot analysis of PEV markers [file 12929_2024_1072_MOESM5_ESM.docx]

*
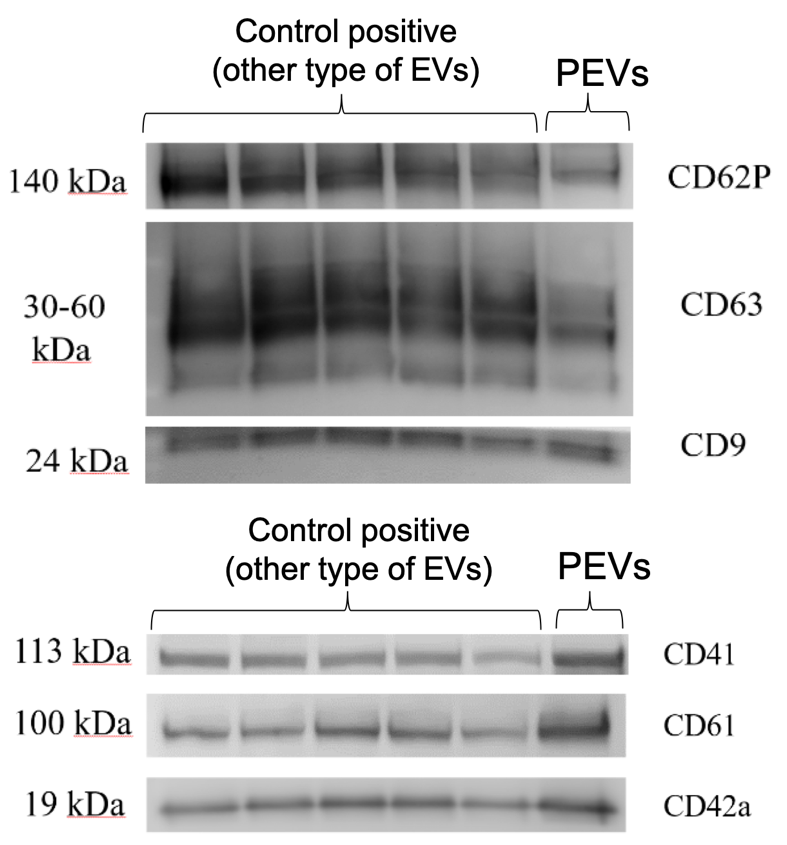
*

FIGURE S2. Western blot analysis of PEV markers. 30 μg of PEV proteins were used. PEVs were found to express EVs markers CD63 and CD9 and platelet markers, CD62P, CD41, CD61, and CD42a. GAPDH was used as an internal control. Abbreviations: Platelet-extracellular vesicles (PEVs).
