# Supplementary material for: Neuroprotective effects of intranasal extracellular vesicles from human platelet concentrates supernatants in traumatic brain injury and Parkinson’s disease models
Source: J Biomed Sci. 2024 Sep 5;31:87. doi: 10.1186/s12929-024-01072-z (PMC11375990; doi:10.1186/s12929-024-01072-z)
Supplement: Supplementary file 6 — Supplementary Material 6. Figure S3. Procoagulant assays [file 12929_2024_1072_MOESM6_ESM.docx]

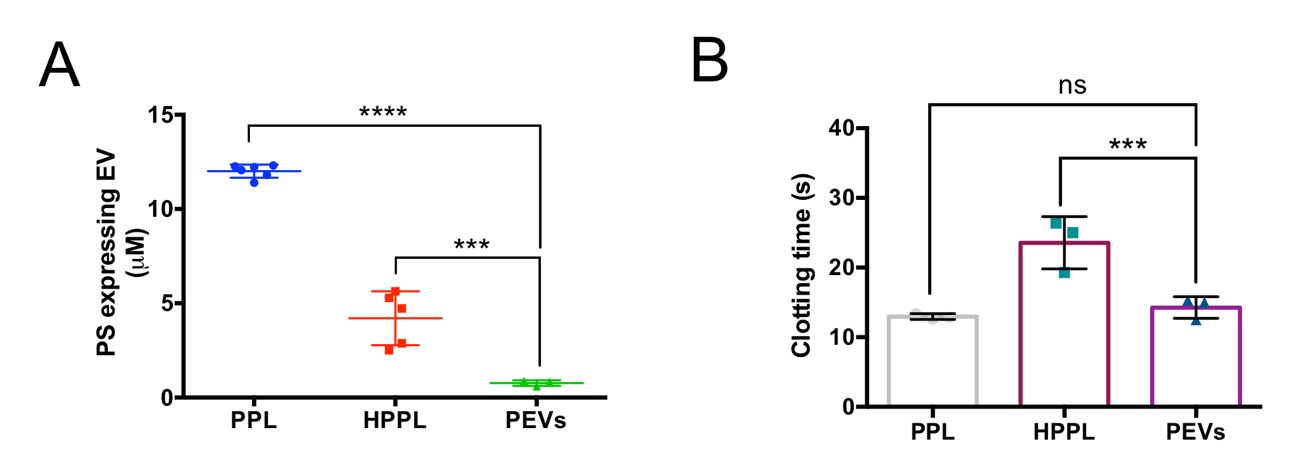


FIGURE S3. Procoagulant assays. (A) Phosphatidylserine (PS)-expressing PEVs. (B) STA-Phospholipids pro-coagulant activity (clotting time). Results are expressed as the means ± SD (N = 3). (***p < 0.001, ****p < 0.0001), compared to PPL and HPPL, ns (no significant). Abbreviations: Platelet-extracellular vesicles (PEVs), Platelet pellet lysate (PPL), Heat-treated platelet pellet lysate (HPPL).
