# Supplementary material for: Neuroprotective effects of intranasal extracellular vesicles from human platelet concentrates supernatants in traumatic brain injury and Parkinson’s disease models
Source: J Biomed Sci. 2024 Sep 5;31:87. doi: 10.1186/s12929-024-01072-z (PMC11375990; doi:10.1186/s12929-024-01072-z)
Supplement: Supplementary file 7 — Supplementary Material 7. Figure S4. PEVs diffusion in the mice brain [file 12929_2024_1072_MOESM7_ESM.docx]

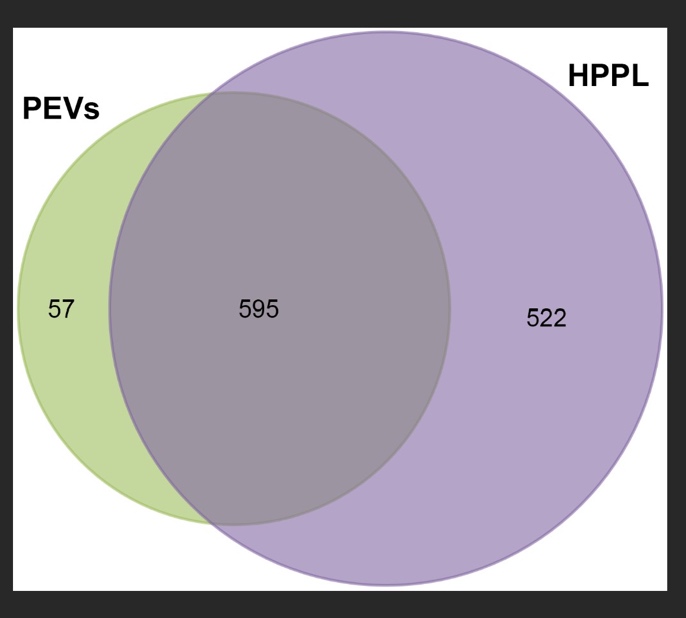


Figure S5. Totals of 1117 and 652 (with a false discovery rate [FDR] of <1%, at the protein level) were identified in HPPL and PEVs, respectively by LC-MS/MS. The Venn diagram illustrates that 595 proteins (90% of the PEV proteins) were also present in HPPL.
