# Supplementary material for: Neuroprotective effects of intranasal extracellular vesicles from human platelet concentrates supernatants in traumatic brain injury and Parkinson’s disease models
Source: J Biomed Sci. 2024 Sep 5;31:87. doi: 10.1186/s12929-024-01072-z (PMC11375990; doi:10.1186/s12929-024-01072-z)
Supplement: Supplementary file 8 — Supplementary Material 8. Figure S5. Venn Diagram of common proteins present in both PEVs and HPPL [file 12929_2024_1072_MOESM8_ESM.docx]

**
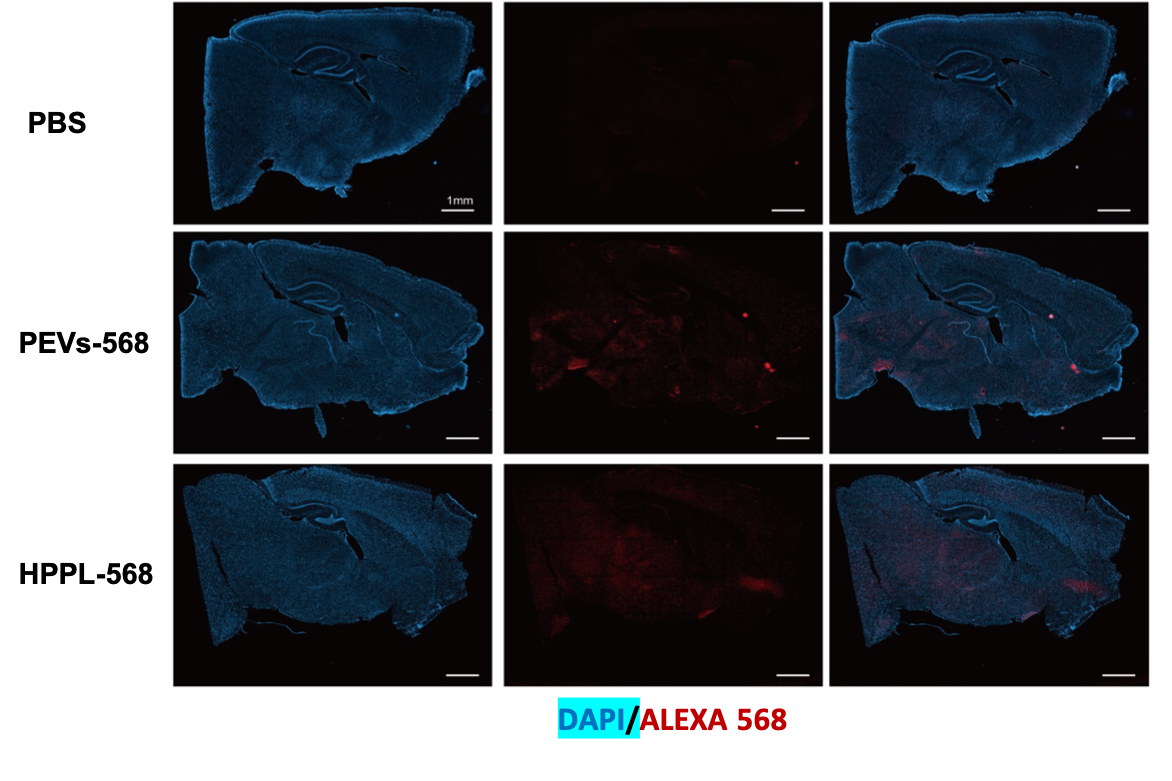
**FIGURE S4. PEVs diffusion in the mice brain. PEVs were observed diffusing through a sagittal section of the mice brain. Detection of PEVs labelled with Alexa Fluor 568 was performed using a fluorescence slide scanner. After 7 hours of intranasal delivery, a distinct red fluorescent spot was visible in the brain slice that received PEVs and HPPL labelling, in contrast to the control with PBS-labeled Alexa Fluor. This diffusion was observed evenly throughout the brain, extending to the cortex, hippocampus, thalamus, and striatum.
